# Temporal-topological properties of higher-order evolving networks

**DOI:** 10.1038/s41598-023-32253-9

**Published:** 2023-04-11

**Authors:** Alberto Ceria, Huijuan Wang

**Affiliations:** Faculty of Electrical Engineering, Mathematics, and Computer Science, Delft University of Technology, Mekelweg 4, 2628 CD Delft, The Netherlands

**Keywords:** Computational science, Complex networks

## Abstract

Human social interactions are typically recorded as time-specific dyadic interactions, and represented as evolving (temporal) networks, where links are activated/deactivated over time. However, individuals can interact in groups of more than two people. Such group interactions can be represented as higher-order events of an evolving network. Here, we propose methods to characterize the temporal-topological properties of higher-order events to compare networks and identify their (dis)similarities. We analyzed 8 real-world physical contact networks, finding the following: (a) Events of different orders close in time tend to be also close in topology; (b) Nodes participating in many different groups (events) of a given order tend to involve in many different groups (events) of another order; Thus, individuals tend to be consistently active or inactive in events across orders; (c) Local events that are close in topology are correlated in time, supporting observation (a). Differently, in 5 collaboration networks, observation (a) is almost absent; Consistently, no evident temporal correlation of local events has been observed in collaboration networks. Such differences between the two classes of networks may be explained by the fact that physical contacts are proximity based, in contrast to collaboration networks. Our methods may facilitate the investigation of how properties of higher-order events affect dynamic processes unfolding on them and possibly inspire the development of more refined models of higher-order time-varying networks.

## Introduction

Interactions among individuals are usually experimentally measured as time-resolved records of face-to-face contacts between couples of people in controlled social setting such as workplaces, hospitals, schools and conferences. These time specific records are thus collected in the form of dyadic interactions, and have been effectively studied in the framework of evolving (temporal) networks, where each link between two nodes is activated only when the node pair interacts^1–3^. The temporal patterns of link activations (or contacts) in real-world networks are far from being fully random nor deterministic^4^. Contacts between a pair of nodes usually occur in bursts of many contacts close in time followed by a long period of inactivity^5^ and the time between two consecutive interactions is usually fat-tailed distributed^6–8^. Such temporal properties of contacts influence the dynamic processes unfolding on the network^9–17^. Despite these tremendous advances in the last decade, studies on temporal networks have traditionally focused on pairwise interactions only. However pairwise interactions can only partially capture interactions among constituents of a system^18,19^. For example, a neuron may receive the output from or send a signal to many different neighbouring neurons^20^, individuals may gather in groups^21^, and scientific collaborations are not limited to couples of authors^22^. Such interactions are named higher-order, to emphasize that they involve more than just a couple of nodes. Benson et al.^23^ showed that a generalization of triadic closure seems to lead the first activation of a given hyperlink. On the other hand, Cencetti et al.^24^ focused on temporal inhomogeneities of activations of the same hyperlink. The focus so far is on the prediction of hyperlink activations^23^ or on pure temporal properties of higher-order events^24^. However, the interplay between temporal and topological properties of higher-order events, e.g. if higher-order events close in time tend to occur also close in topology, remains far from well understood. Hence, this work aims to systematically characterize the relation between temporal and topological properties of higher-order events to compare higher-order temporal networks. Inspired by our recent work that characterizes temporal and topological properties of dyadic interactions in temporal networks^25^, we redesign the characterization method for higher-order events. In particular, we are going to explore such properties from three perspectives: (1) The interrelation between the distance in topology and the temporal delay of events, (2) Their correlation or overlap in topological location. (3) The temporal correlation of local events that overlap in component nodes. In order to compare real-world networks with different sizes, we design null models where temporal and topological properties of events of an arbitrary order are systematically destroyed or preserved. We applied our methods to 8 real-world physical contact networks and 5 collaboration networks. We show that, in physical contacts, events of different orders with short temporal delay tend to be close in topology too. We then investigate the correlation of events in topology and discover that events of different orders are likely to overlap in component nodes. In particular, nodes who participate in many different groups (events) of a given order are likely to be involved in many different groups (events) of another order. Individuals do not reduce their number of interactions of one order due to frequent interactions of another order. Finally, we show that those local events that overlap in component nodes are correlated in time, which supports the finding that events close in time are also close in topology. In collaboration networks, we observe that events also overlap in component nodes. However, the correlation between topological distance and temporal delay of events are usually either weak or absent. Coherently, in collaboration networks, the temporal correlation of local events that overlap in component nodes is almost absent. Such differences between physical contacts and collaboration networks may be due to the fact that physical interactions are partly driven by proximity, so that a set of individuals close to each other tend to interact close in time among (subsets of) them.

Our methods can be applied to compare real-world higher-order networks and to investigate how the properties of their events affects the dynamic processes unfolding on them. More realistic models of higher-order evolving networks can be further developed to reproduce specific properties of the higher-order interactions observed in this paper.

## Definitions

### Higher-order evolving networks

Time-varying social interactions or contacts have been mostly measured pairwise and studied with the formalism of (pairwise) temporal networks. A temporal network observed at discrete time within [0, *T*) can be described by ${\mathscr {G}} = ({\mathscr {N}}, {\mathscr {C}})$, where ${\mathscr {N}}$ is the set of nodes or individuals, ${\mathscr {C}}$ is the set of pairwise interactions. If node *u* and *v* have a contact at time step $0\le t \le T-1$, $(\ell ,t) \in {\mathscr {C}}$, where $\ell = \ell (u,v)$ is the link connecting the pair of nodes between which the contact occurs. The contact $(\ell (u,v),t)$ can be regarded as the activation of the link $\ell (u,v)$ at time *t*. This traditional temporal network representation records social contacts as a set of pair-wise interactions. However, individuals may gather in larger groups, so that more than two people interact with each other at the same time. For example, an interaction (*h*(*i*, *j*, *k*), *t*) among three nodes at time *t* is usually measured and recorded as three pair-wise interactions $(\ell (i,j),t)$, $(\ell (j,k),t)$ and $(\ell (i,k),t)$. Social interactions can be more precisely represented as a higher-order evolving network ${\mathscr {H}} = ({\mathscr {N}},{\mathscr {E}})$ (or temporal hypergraph, following the definition of Cencetti et al.^24^), where ${\mathscr {E}}$ is the set of events of arbitrary orders. Such group interaction or higher-order event $(h(u_1,\dots u_d),t)$ can be regarded as the activation of the corresponding hyperlink $h(u_1,\dots u_d)$ at *t*. The size or order of the interaction is *d*, where *d* is the size of the group. The pairwise time aggregated network of a traditional pairwise temporal network is $G = ({\mathscr {N}},\Lambda )$, where any couple of nodes (*i*, *j*) is connected by a link $\ell (i,j) \in \Lambda$ if $\ell (i,j)$ has been active at least once during the entire observation time [0, *T*). Consistently, the higher-order time aggregated network is $H = ({\mathscr {N}},{\mathscr {L}})$, where any set $\{u_1,\dots u_d\}$ of *d* nodes are connected by a hyperlink $h(u_1,\dots u_d) \in {\mathscr {L}}$ with size *d* if $h(u_1,\dots u_d)$ has been activated at least once. The activity of each hyperlink *h* can be represented by a time series $X_{h} = \{x_{h}(t), 0\le t < T\}$ where $x_{h}(t) = 1$ only if the hyperlink *h* is active at time t, i.e., $e=(h,t) \in {\mathscr {E}}$.

### Temporal and topological distance of events

The temporal distance or delay between two events $e_1 = (h_1, t)$ and $e_2 = (h_2, s)$ is ${\mathscr {T}}(e_1, e_2)=|t-s|$.

The topological distance, also called hop-count, between two nodes on a pair-wise static network is the number of links contained in the shortest path between these two nodes. We define the topological distance $\eta (e_1,e_2)$ between two events $e_1 = (h_1, t)$ and $e_2 = (h_2, s)$ as the topological distance between the corresponding two hyperlinks $h_1$ and $h_2$, which is further defined as follows. The distance between the same hyperlink is zero, e.g., $\eta ((h_1,t),(h_1,s)) = 0$. The distance between two different hyperlinks $h(u_1,\dots ,u_d)$ and $h(v_1,\dots ,v_{d'})$ with size *d* and $d'$, respectively, follows

1 $$\begin{aligned} \eta ((h(u_1,\dots ,u_d),t),(h(v_1,\dots ,v_{d'}),s)) = min_{u\in \{ u_1,\dots ,u_d\}, v \in \{v_1,\dots ,v_{d'}\}}(\delta (u,v)+1) \end{aligned}$$

where $\delta (u,v)$ is the distance or hop-count between node *u* and *v* on the unweighted pairwise time aggregated network *G*. The distance between two events is thus one plus the minimal distance between two component nodes from the two events respectively. For example, the distance between events $e_1 = (h(i,j,k),t)$ and $e_2 = (h(i,m,n),s)$ is $\eta (e_1,e_2) = 1$.

### Network randomization-control methods

To detect non-trivial temporal and topological patterns of events, we compare properties obtained from real-world higher-order temporal networks with those of designed null models. We generalize the randomized reference models of pairwise evolving networks which gradually preserve and destroy temporal and topological properties of pairwise interactions^25–27^ for higher-order temporal networks. Given a higher-order evolving network ${\mathscr {H}}$ and any given order *d* of events, we introduce 3 randomized null models ${\mathscr {H}}^1_d$, ${\mathscr {H}}^2_d$ and ${\mathscr {H}}^3_d$ which systematically randomize order *d* events only, without changing events of any other order $d'\ne d$. We denote as ${\mathscr {E}}_d$ the set of events with the same size *d*. Randomized network ${\mathscr {H}}^1_{d}$ is obtained by randomly re-shuffling the time stamps of the events in ${\mathscr {E}}_d$, without changing the topological locations of these events. This randomization does not change the total number of activations of each hyperlink, nor the probability distribution of the topological distance of two randomly selected events. Null model ${\mathscr {H}}^1_{d}$ randomizes the time stamps of order *d* events. As a consequence, the distribution of the inter-event time of order *d* events, i.e., the time between two consecutive activations of a random order *d* hyperlink, in ${\mathscr {H}}^1_{d}$ tends to be less heterogeneous than that in ${\mathscr {H}}$. As mentioned above, the activations of a given hyperlink *h* can be represented by a time series $X_{h}$. The randomized network ${\mathscr {H}}^2_d$ is obtained by iteratively swapping the time series of two randomly selected order *d* hyperlinks . In ${\mathscr {H}}^2_d$, the inter-event time distribution of order *d* events is preserved as in the original network ${\mathscr {H}}$, while the time series of activations of a given order *d* hyperlink are independent from its topological location. The third randomized network ${\mathscr {H}}^3_d$ is obtained by swapping the activity time series of two randomly selected order *d* hyperlinks with the same total number of activations. This randomization does not change the number of activations of any hyperlink, the distribution of the topological distance of two random events, nor the inter-event (order *d* events) time distribution. The pairs of order *d* hyperlinks with the same number of events can be few in number in real-world temporal networks, such that the difference between a real-world network and its randomized network ${\mathscr {H}}_{d}^3$ is small. This is especially the case when the order *d* is large, thus the number of hyperlinks is small. These three randomized models preserve the unweighted higher-order time aggregated network *H* and the probability distribution of the temporal distance of two random events of size *d*.

## Datasets

We will apply our method to 13 real-world datasets of human physical interactions and scientific collaborations. The first 8 datasets are collections of face-to-face interactions at a distance smaller than 2 m in several social contexts such as conferences (HT2009, SFHH), hospital, primary school (PS), high schools (HS2012,HS2013), workplace (WP2) and museum (Infectious). Face-to-face interactions are recorded as a set of pair-wise interactions. Based on them, we deduce group interactions, by promoting each set of $$\left( {\begin{array}{c}d\\ 2\end{array}}\right)$$ dyadic interactions occurring at the same time and forming a fully connected clique of *d* nodes to an event of size *d*. Since a clique of order *d* contains all its sub-cliques of order $d'<d$, only the maximal clique is promoted to a higher-order event, whereas sub-cliques are ignored. For example, 3 pairwise contacts $(\ell (i,j),t),\ (\ell (j,k),t)$ and $(\ell (i,k),t)$ occurring at the same time *t* are regarded as a single event of order 3 i.e., (*h*(*i*, *j*, *k*), *t*) without any order 2 event. This method has been already used by Cencetti et al.^24^. to deduce higher-order interactions from datasets of human face-to-face interactions. We further preprocess these datasets by removing nodes which are not connected to the largest connected component in the pairwise time-aggregated network. We also remove long periods of inactivity, when no event occurs in the network. Such periods usually correspond, e.g., to night and weekends, and are recognized as outliers in the inter-event time distribution of the time series which records the total number of events per timestamp. Such data pre-processing method has also been used in our recent work^25^. The other 5 higher-order collaborations networks are obtained based on scientific papers recorded in the arxiv in various fields: lattice high energy physics (hep-lat), theoretical nuclear physics (nucl-th), quantitative biology (q-bio), quantitative finance (q-fin) and quantum physics (quant-ph). In a collaboration network, each node represents an author, and an event of order *d* occurrs at time t if a paper co-authored by *d* authors is published at t. Assigning papers to the correct authors is not easy. The same author can be named differently, e.g., using the full or initial of the first name and typographic errors may be present. Thus, we applied standard text preprocessing methods to authors’ name, and we identify each author by the initials of their first names, together with their surname according to the method of Newman et al.^28^. The total number of events of each order in each real-world temporal network is shown in Figs. S1 and S2 in Supplementary Material. In each dataset, the number of events with order $2\le d \le 4$ is not negligible; however events with an order larger than 4 are rare (if not absent) in most of the physical contact datasets. Details of the datasets after preprocessing are given in Table 1.

**Table 1:** Basic features of the empirical higher-order time-evolving networks after data processing.

Network	$|{\mathscr {N}}|$	$|{\mathscr {L}}|$	$|{\mathscr {E}}|$	*T*	*dt*	Contact type
Primary school (PS)	242	12,704	106,877	3099	20 s	Physical
High school 2013 (HS2013)	327	7818	172,031	7371	20 s	Physical
Hypertext 2009 (HT2009)	113	2434	19,037	7227	20 s	Physical
Infectious (infectious)	410	3350	14,275	1422	20 s	Physical
Workplace 2015 (WP2)	217	4909	73,820	20,947	20 s	Physical
SFHH conference (SFHH)	403	10,541	54,306	3800	20 s	Physical
Hospital (hospital)	75	1825	27,835	16,027	20 s	Physical
High school 2012 (HS2012)	180	2645	42,105	14,115	20 s	Physical
High energy physics, lattice (hep-lat)	10,598	11,588	18,267	10,809	1 d	Collaboration
Nuclear physics, theory (nucl-th)	25,246	27,094	39,511	10,620	1 d	Collaboration
Quantitative biology (q-bio)	45,645	22,978	25,973	10,704	1 d	Collaboration
Quantitative finance (q-fin)	7509	6192	7577	9027	1 d	Collaboration
Quantum physics (quant-ph)	56,036	70,119	88,769	10,600	1 d	Collaboration

The number of nodes ($|{\mathscr {N}}|$), the number of hyperlinks ($|{\mathscr {L}}|$), the total number of events ($|{\mathscr {E}}|$), the length of the observation time window in time steps (*T*), the time resolution or duration of each time step (*dt*) in seconds or days and the contact type are shown.

## Characterizing temporal-topological properties of networks

In this section we introduce a systematic characterization method of higher-order temporal networks. We characterize the temporal and topological properties of events from three different perspectives. First, we analyze the interrelation between the temporal and topological distance of two arbitrary events of different orders. Then, we study the topological correlation of events, i.e., how events of different orders overlap in component nodes. Finally, we introduce a method to characterize the temporal correlation of events occurring close in topology.

### Correlation of temporal and topological distance of events

**Figure 1:**
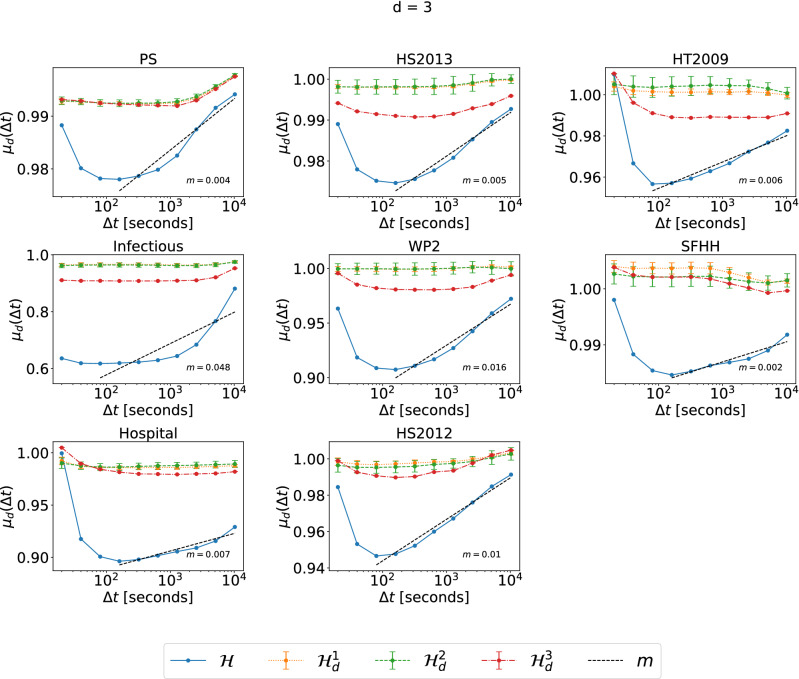
The normalized average topological distance $\mu _d(\Delta t) =\frac{E[\eta (e,e') | {\mathscr {T}} (e,e') < \Delta t,\ e\in {\mathscr {E}}_d,\ e' \in {\mathscr {E}}\setminus {\mathscr {E}}_d ]}{E[\eta (e,e')|\ e\in {\mathscr {E}}_d,\ e' \in {\mathscr {E}}\setminus {\mathscr {E}}_d]}$, between an order $d=3$ event and an event of a different order, in each physical contact network and its corresponding three randomized null models ${\mathscr {H}}^1_d$ (yellow), ${\mathscr {H}}^2_d$ (green) and ${\mathscr {H}}^3_d$ (red), which preserve or destroy specific properties of order $d=3$ events. $\lim _{\Delta t\rightarrow \infty } E[\eta (e,e') | {\mathscr {T}} (e,e') < \Delta t,\ e\in {\mathscr {E}}_d,\ e' \in {\mathscr {E}}\setminus {\mathscr {E}}_d ] =E[\eta (e,e')|\ e\in {\mathscr {E}}_d,\ e' \in {\mathscr {E}}\setminus {\mathscr {E}}_d]$ for any *d*. The horizontal axes are presented in logarithmic scale. The dashed line in each figure corresponds to the linear fit (with slope *m*) of $\mu _{d}(\Delta t)$ as a function of $log_{10}(\Delta t)$ in ${\mathscr {H}}$, for the part that the curve has an increasing trend. For each dataset, the results of the three corresponding randomized models are obtained from 10 independent realizations.

**Figure 2:**
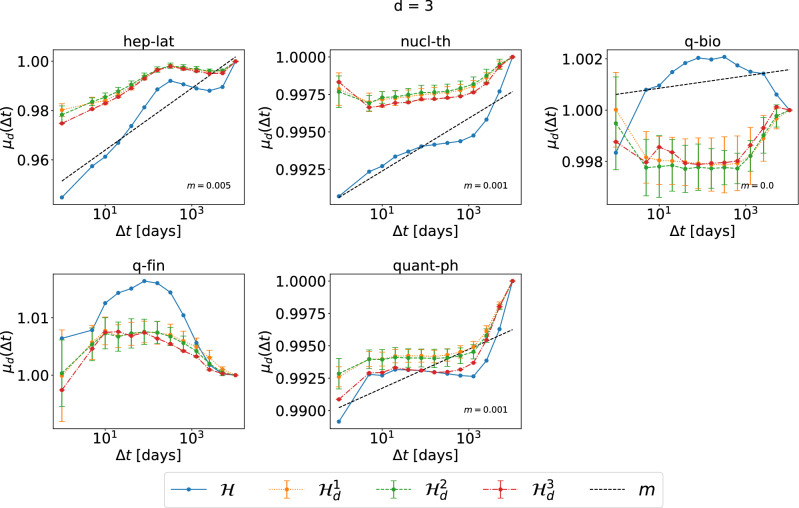
The normalized average topological distance $\mu _d(\Delta t) =\frac{E[\eta (e,e') | {\mathscr {T}} (e,e') < \Delta t,\ e\in {\mathscr {E}}_d,\ e' \in {\mathscr {E}}\setminus {\mathscr {E}}_d ]}{E[\eta (e,e')|\ e\in {\mathscr {E}}_d,\ e' \in {\mathscr {E}}\setminus {\mathscr {E}}_d]}$, between an order $d=3$ event and an event of a different order, in each collaboration network and its corresponding three randomized null models ${\mathscr {H}}^1_d$ (yellow), ${\mathscr {H}}^2_d$ (green) and ${\mathscr {H}}^3_d$ (red), which preserve or destroy specific properties of order $d=3$ events. $\lim _{\Delta t\rightarrow \infty } E[\eta (e,e') | {\mathscr {T}} (e,e') < \Delta t,\ e\in {\mathscr {E}}_d,\ e' \in {\mathscr {E}}\setminus {\mathscr {E}}_d ] =E[\eta (e,e')|\ e\in {\mathscr {E}}_d,\ e' \in {\mathscr {E}}\setminus {\mathscr {E}}_d]$ for any *d*. The horizontal axes are presented in logarithmic scale. The dashed line in each figure corresponds to the linear fit (with slope *m*) of $\mu _{d}(\Delta t)$ as a function of $log_{10}(\Delta t)$ in ${\mathscr {H}}$, for the part that the curve has an increasing trend. For each dataset, the results of the three corresponding randomized models are obtained from 10 independent realizations.

In this subsection we investigate how temporal and topological distance of events are related to each other. Specifically, we aim to understand to what extent events close in time are also close in topology. In our previous work^25^, we considered all interactions in a temporal network as pairwise interactions alone and found in real-world physical and virtual contact networks that pairwise interactions that are close in time tend to be close in topology (in the pairwise time aggregated network). Here, we generalize the method of characterizing the relation between topological and temporal distance of two dyadic interactions to that of two higher-order events with different orders. In this analysis, normalizations in topological distance and randomizations in networks have been applied so that we can compare real-world temporal networks with different properties in e.g., the number of nodes and contacts. We take order $d= 3$ as an example to illustrate our method and observations. In Figs. 1 and 2 we investigate the average topological distance $E[\eta [(e,e')|{\mathscr {T}}(e,e')<\Delta t,e \in {\mathscr {E}}_d,\ e' \in {\mathscr {E}}\setminus {\mathscr {E}}_d]$ between two events $(e,e')$ with different orders $d\ne d'$, given that their temporal distance is smaller than $\Delta t$ in physical contact and collaboration networks, respectively. In physical contact networks (Fig. 1), we observe in general an increasing trend of the normalized average topological distance $\mu _d(\Delta t) =\frac{E[\eta (e,e') | {\mathscr {T}} (e,e') < \Delta t,\ e\in {\mathscr {E}}_d,\ e' \in {\mathscr {E}}\setminus {\mathscr {E}}_d ]}{E[\eta (e,e')|\ e\in {\mathscr {E}}_d,\ e' \in {\mathscr {E}}\setminus {\mathscr {E}}_d]}$ between between events of different orders with their conditional temporal distance $\Delta t$, except that the topological distance decreases with $\Delta t$ when $\Delta t$ is small, approximately when $\Delta t\le 100s$ . Usually, events of different orders that occur relatively close in time tend to be also close in topology. The decrease of the average distance $\mu _d(\Delta t)$ with $\Delta t$ when $\Delta t$ is small is introduced by the way how higher-order physical contact networks are constructed. In these networks higher-order events are inferred from their contact records, so that if a higher-order event that involves a set of *d* nodes occur at a given timestamp, no event of an order $d'$ smaller than *d* involving only a subset of these *d* nodes can occur at the same timestamp. This explains why as $\Delta t$ decreases further when it is small, the topological distance $\mu _d(\Delta t)$ does not decrease anymore. This is not the case in collaboration networks, where when a group of scientists collaborate in a paper, a sub-group could co-author another paper at the same time. Accordingly, we do not observe the decreasing trend of the $\mu _d(\Delta t)$ with $\Delta t$ when $\Delta t$ is small in collaboration networks. Besides this initial decreasing trend, we observe an increasing trend of $\mu _d(\Delta t)$ between events with their conditional temporal distance in every physical contact networks, but this is generally much less evident in collaboration networks. The slope of the increase of $\mu _d(\Delta t)$ with the conditional temporal distance $\Delta t$ indicates the relative strength of temporal-topological correlation of events. In Figs. 1 and 2 we show the slope of the linear fit of $\mu _d(\Delta t)$ as a function of $log_{10}(\Delta t)$ for the part of the curve that has an increasing trend. In physical contacts, the highest slopes are observed in Infectious and Workplace (WP2) networks. Moreover, in each dataset we observe an increasing trend with slope larger than 0. In contrast, this slope is small around zero in the corresponding randomized network ${\mathscr {H}}_d^1$, ${\mathscr {H}}_d^2$ and ${\mathscr {H}}_d^3$. This means the set of activity time series of each order 3 hyperlink of a higher-order network ${\mathscr {H}}$, which is preserved in the corresponding randomized network ${\mathscr {H}}_d^2$ and ${\mathscr {H}}_d^3$ does not contribute to the correlation between topological and temporal distance of events of different orders.

Differently, in collaboration networks, the increasing trend is usually either very weak (nucl-th, quant-ph) or absent (q-bio and q-fin), with the only exception of hep-lat dataset. The temporal-topological correlation of events tends to disappear in collaboration networks.

Conclusions drawn from the discussion of Figs. 1 and 2 hold for the other orders $d = 2$ (see Figs. S5 and S6 in Supplementary Material) and $d=4$ (see Figs. S7 and S8 in Supplementary Material). The only exceptions are observed in datasets HT2009 and WP2 when $d=4$: in this case indeed the trend of $\mu _d(\Delta t)$ in three randomized reference models seems to partially re produce the increasing trend observed in ${\mathscr {H}}$. This is likely due to the low number of hyperlinks of order 4 in these two networks.

**Figure 3:**
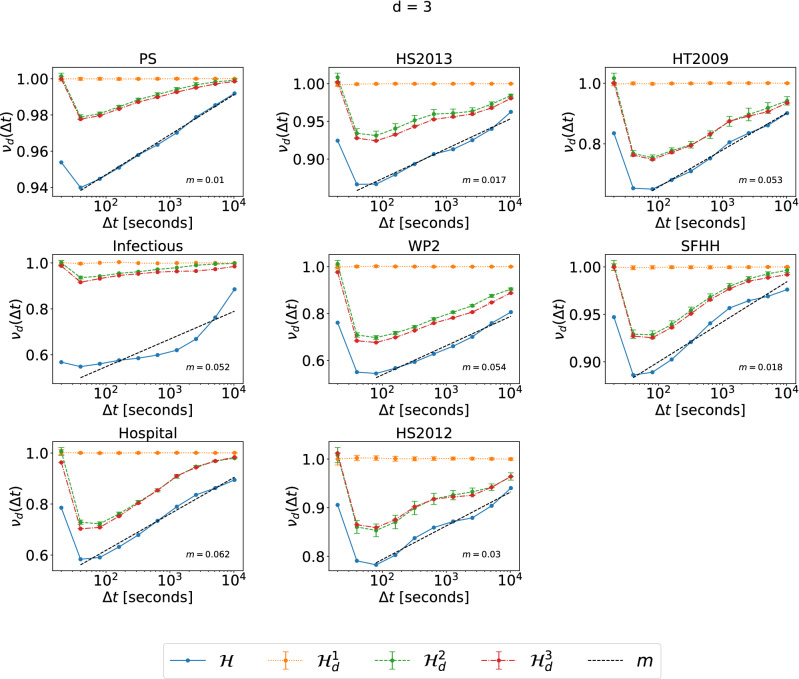
The normalized average topological distance $\nu _d(\Delta t) =\frac{E[\eta (e,e') | {\mathscr {T}} (e,e') < \Delta t,\ e,\ e' \in {\mathscr {E}}_d]}{E[\eta (e,e')|\ e,\ e'\in {\mathscr {E}}_d]}$, between two order $d=3$ events, in each physical contact network and its corresponding three randomized null models ${\mathscr {H}}^1_d$ (yellow), ${\mathscr {H}}^2_d$ (green) and ${\mathscr {H}}^3_d$ (red), which preserve or destroy specific properties of order $d=3$ events. $\lim _{\Delta t\rightarrow \infty } E[\eta (e,e') | {\mathscr {T}} (e,e') < \Delta t,\ e,\ e' \in {\mathscr {E}}_d ] =E[\eta (e,e')|\ e,\ e'\in {\mathscr {E}}_d]$ for any *d*. The horizontal axes are presented in logarithmic scale. The dashed line in each figure corresponds to the linear fit (with slope *m*) of $\nu _{d}(\Delta t)$ as a function of $log_{10}(\Delta t)$ in ${\mathscr {H}}$, for the part that the curve has an increasing trend. For each dataset, the results of the three corresponding randomized models are obtained from 10 independent realizations.

**Figure 4:**
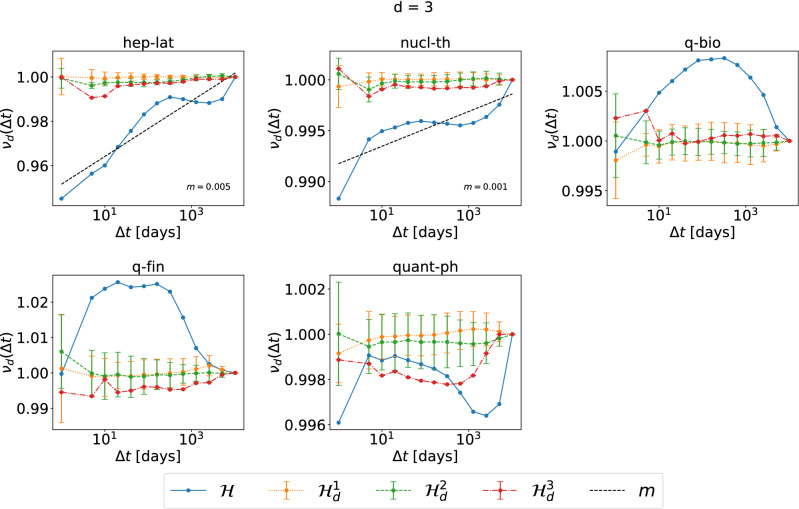
The normalized average topological distance $\nu _d(\Delta t) =\frac{E[\eta (e,e') | {\mathscr {T}} (e,e') < \Delta t,\ e,\ e' \in {\mathscr {E}}_d]}{E[\eta (e,e')|\ e,\ e'\in {\mathscr {E}}_d]}$, between two order $d=3$ events, in each collaboration network and its corresponding three randomized null models ${\mathscr {H}}^1_d$ (yellow), ${\mathscr {H}}^2_d$ (green) and ${\mathscr {H}}^3_d$ (red), which preserve or destroy specific properties of order $d=3$ events. $\lim _{\Delta t\rightarrow \infty } E[\eta (e,e') | {\mathscr {T}} (e,e') < \Delta t,\ e,\ e' \in {\mathscr {E}}_d ] =E[\eta (e,e')|\ e,\ e'\in {\mathscr {E}}_d]$ for any *d*. The horizontal axes are presented in logarithmic scale. The dashed line in each figure corresponds to the linear fit (with slope *m*) of $\nu _{d}(\Delta t)$ as a function of $log_{10}(\Delta t)$ in ${\mathscr {H}}$, for the part that the curve has an increasing trend. For each dataset, the results of the three corresponding randomized models are obtained from 10 independent realizations.

We focus on the analysis of events of different orders. We have also analyzed events of the same order and obtain similar observations. As an example, Figs. 3 and 4, show the normalized average topological distance $\nu _d(\Delta t) = \frac{E[\eta (e,e') | {\mathscr {T}} (e,e') < \Delta t,\ e,\ e' \in {\mathscr {E}}_d]}{E[\eta (e,e')|\ e,\ e'\in {\mathscr {E}}_d]}$ of events of the same order $d=3$ with a temporal delay smaller than $\Delta t$. The temporal-topological correlation is observed in physical contact networks but not collaboration networks. In contrast to events of different orders, in physical contacts, events of the same order demonstrate similar temporal-topological correlation in randomized networks ${\mathscr {H}}^2_d$ and ${\mathscr {H}}^3_d$ as in the corresponding real-world network ${\mathscr {H}}$, reflected the similar slope of the increase of the topological distance with $\Delta t$ in these three networks. Randomized network ${\mathscr {H}}^2_d$ and ${\mathscr {H}}^3$ preserve the same set of activity time series of each single order *d* hyper link. The burstiness property, i.e. the frequent activation of the same hyperlink within a short time followed by a long resting period of an activity time series contributes to the temporal-topological correlation observed in real-world physical networks. These conclusions hold also for the analysis for orders $d = 2$ (Figs. S9 and S10 in Supplementary Material) and 4 (Figs. S11 and S12 in Supplementary Material). The only exception is that no evident increase of $\nu _d(\Delta t)$ with $\Delta t$ is observed when $d=4$ in Workplace and Hypertext 09, likely due to the low number of order $d=4$ events observed in these two networks. In this work, we focus on the analysis of events of different orders, whose temporal-topological correlation cannot be explained by the burstiness of the activations of each hyperlink.

### Topological correlation of events with different orders

**Figure 5:**
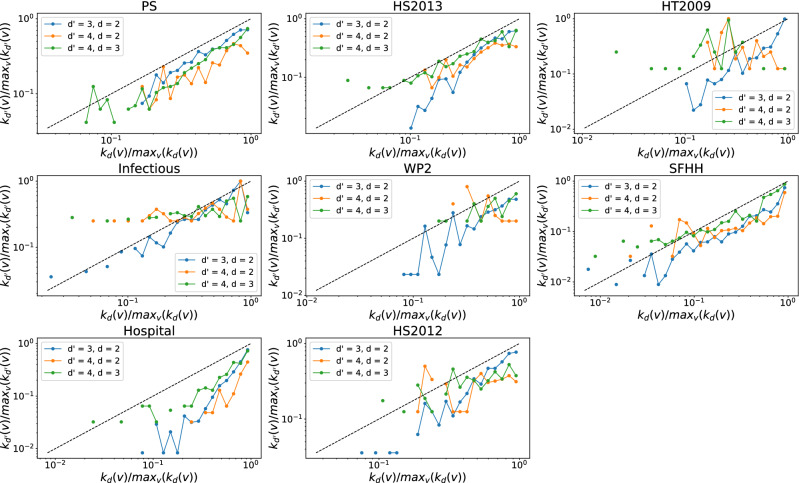
The $d'$-degree $k_{d'}(v)$ versus the the *d*-degree $k_{d}(v)$ of a node *v* when $(d',d)$ is equal to (3,2) (blue line), (4,2) (yellow line) and (4,3) (green line) respectively in each physical contact network. Each axis (e.g., $k_{d}(v)$) has been normalized by its maximum (e.g., $max_{v}(k_{d}(v))$). Only nodes whose *d*-degree and $d'$-degree are both non-zero are considered. The dashed line represent the reference case $\frac{k_{d'}(v)}{max_{v}(k_{d'}(v))} = \frac{k_{d}(v)}{max_{v}(k_{d}(v))}$. Note that both axes are presented in logarithmic scales. In total 30 logarithmic bins are split for horizontal axis.

**Figure 6:**
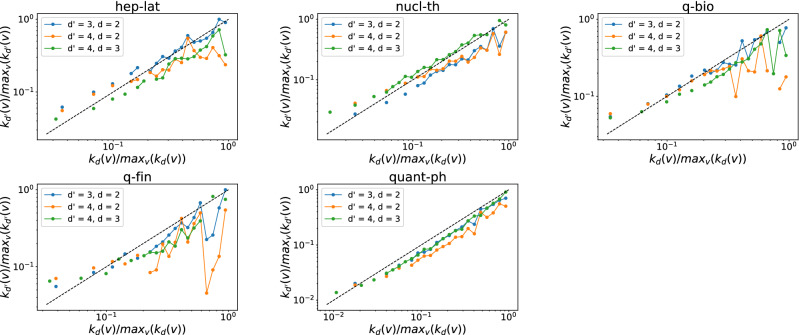
The $d'$-degree $k_{d'}(v)$ versus the the *d*-degree $k_{d}(v)$ of a node *v* when $(d',d)$ is equal to (3,2) (blue line), (4,2) (yellow line) and (4,3) (green line) respectively in each collaboration network. Each axis (e.g., $k_{d}(v)$) has been normalized by its maximum (e.g., $max_{v}(k_{d}(v))$). Only nodes whose *d*-degree and $d'$-degree are both non-zero are considered. The dashed line represent the reference case $\frac{k_{d'}(v)}{max_{v}(k_{d'}(v))} = \frac{k_{d}(v)}{max_{v}(k_{d}(v))}$. Note that both axes are presented in logarithmic scales. In total 30 logarithmic bins are split for horizontal axis.

To better understand the observed correlation between temporal and topological distance of events, we explore further whether higher-order events overlap in component nodes (correlation in topology) in this subsection and whether events that overlap in topology are correlated in time in the next subsection. Higher-order events that overlap in component nodes and occur close in time may partially explain the observed temporal and topological correlation between events. Would a node that belongs to many hyperlinks of order *d*, also be connected to many hyperlinks of order $d'\ne d$? To investigate this question, we examine the number of hyperlinks of each order that a node belongs to in the unweighted higher-order time aggregated network. The total number of order *d* hyperlinks that the node *v* is connected to, denoted as $k_d(v)$, is also called the *d*-degree of node *v*. In Figs. 5, 6, we compare the *d*-degree and the $d'$-degree of a node when $(d',d)$ is equal to (3,2), (4,2) and (4,3) respectively in each physical contact (collaboration) network. All three randomized networks ${\mathscr {H}}^1_d$, ${\mathscr {H}}^2_d$ and ${\mathscr {H}}^3_d$ have the same higher-order time-aggregated unweighted network as the corresponding real-world network ${\mathscr {H}}$. Hence, the $d$-degree and $d'$-degree of each node remain the same in the randomized networks as in the real-world network. We focus on the case when $(d',d)$ is equal to (3,2), as an example. We observe that the $d'$-degree of a node is an increasing function of the *d*-degree of the node in every considered collaboration and physical contact networks. Hence, a node that participates in many groups of order 3, tends to involve in many groups of order 2. When $(d',d)$ equals to (4,2) and (4,3), such trend is less evident in physical networks (especially in WP2, HS2012, Infectious and HT2009) and remains evident in collaboration networks. This is likely because the number of order 4 hyperlinks is generally low (see Fig. S3 in Supplementary Material) in physical contact networks, but not in collaboration networks (see Fig. S4 in Supplementary Material).

**Figure 7:**
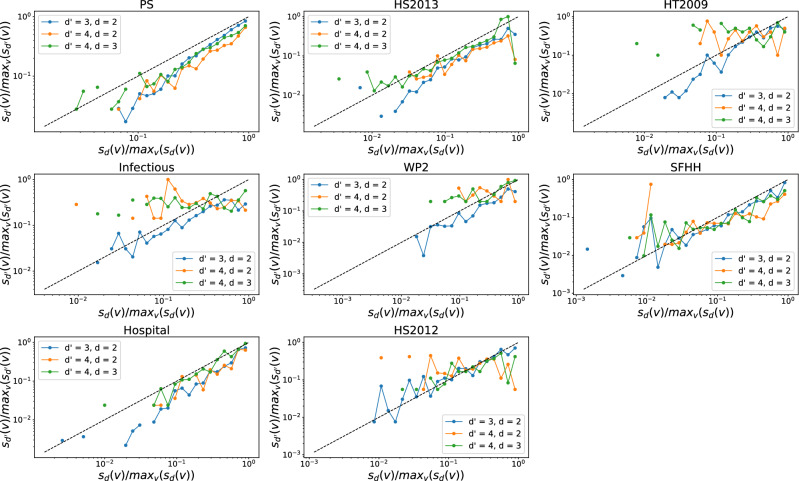
The $d'$-strength $s_{d'}(v)$ versus the the *d*-strength $s_{d}(v)$ of a node *v* when $(d',d)$ is equal to (3,2) (blue line), (4,2) (yellow line) and (4,3) (green line) respectively in each physical contact network. Each axis (e.g., $s_{d}(v)$) has been normalized by its maximum (e.g., $max_{v}(s_{d}(v))$). Only nodes whose *d*-strength and $d'$-strength are both non-zero are considered. The dashed line represent the reference case $\frac{s_{d'}(v)}{max_{v}(s_{d'}(v))} = \frac{s_{d}(v)}{max_{v}(s_{d}(v))}$, where $d'$-strength is a linear function of the *d*-strength of nodes. Note that both axes are presented in logarithmic scales. In total 30 logarithmic bins are split for horizontal axis.

**Figure 8:**
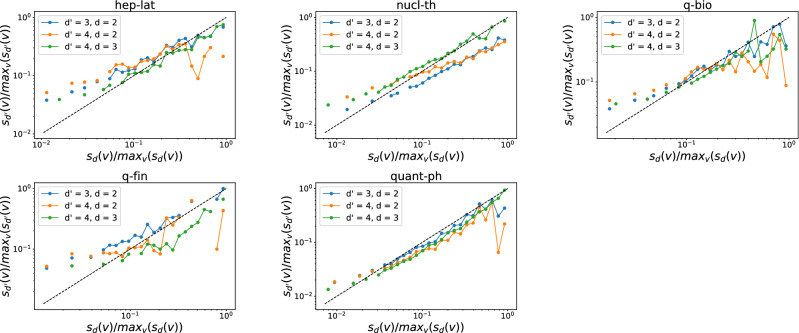
The $d'$-strength $s_{d'}(v)$ versus the the *d*-strength $s_{d}(v)$ of a node *v* when $(d',d)$ is equal to (3,2) (blue line), (4,2) (yellow line) and (4,3) (green line) respectively in each collaboration network. Each axis (e.g., $s_{d}(v)$) has been normalized by its maximum (e.g., $max_{v}(s_{d}(v))$). Only nodes whose *d*-strength and $d'$-strength are both non-zero are considered. The dashed line represent the reference case $\frac{s_{d'}(v)}{max_{v}(s_{d'}(v))} = \frac{s_{d}(v)}{max_{v}(s_{d}(v))}$, where $d'$-strength is a linear function of the *d*-strength of nodes. Note that both axes are presented in logarithmic scales. In total 30 logarithmic bins are split for horizontal axis.

Furthermore, we investigate whether a node that involves in many order *d* events tends to join many order $d'$ interactions. The number of order *d* events that a node *v* is involved in, denoted by $s_d(v)$, is also called the *d*-strength of node *v*. The $d$-strength of a node is actually the sum of the weights of order *d* hyperlinks that a node belong to in the weighted higher-order time aggregated network. The weight of each hyperlink represents the number of events/activations of the hyperlink. Similar to our analysis of the *d*-degree and $d'$-degree of node, we find the *d*-strength and $d'$-strength of a node are also positively correlated when $(d',d)$ equal to (3,2) in each temporal network, as shown in Figs. 7 and 8. This trend is less evident only in physical contacts that have few order 4 events, when $(d',d)$ is equal to (4,3) and (4,2). This suggests that an individual’s large number of interactions of one order would not reduce his or her number of events of another order. Individuals tend to be consistently active or inactive in events across orders.

**Figure 9:**
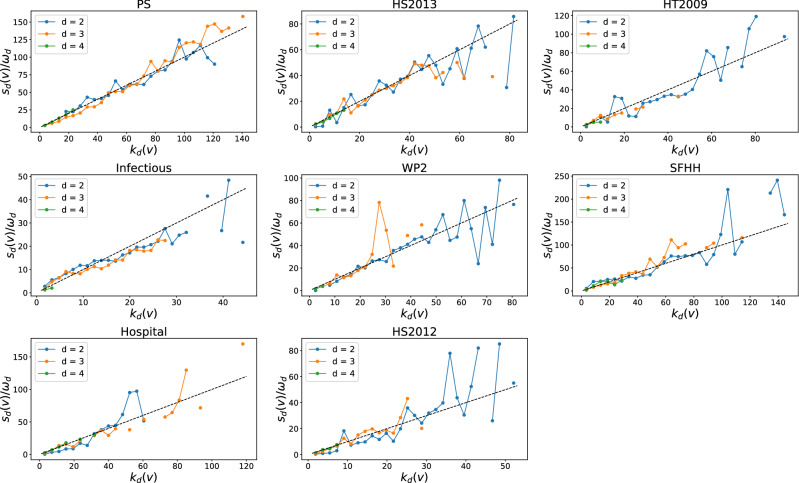
The *d*-strength $s_{d}(v)$ versus the the *d*-degree $k_{d}(v)$ of a node *v* when *d* is equal to 2 (blue line), 3 (yellow line) and 4 (green line) respectively in each physical contact network. The vertical axis is normalized by the average number $\omega_d$ of activations of a hyperlink of order $d$. The dashed line represent the reference case $s_{d}(v) = \omega _d*k_{d}(v)$. In total 30 linear bins are split for horizontal axis.

**Figure 10:**
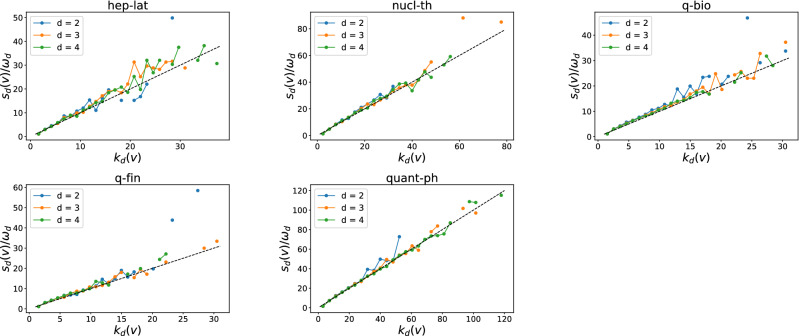
The *d*-strength $s_{d}(v)$ versus the the *d*-degree $k_{d}(v)$ of a node *v* when *d* is equal to 2 (blue line), 3 (yellow line) and 4 (green line) respectively in each collaboration network. The vertical axis is normalized by the average number $\omega_d$ of activations of a hyperlink of order $d$. The dashed line represent the reference case $s_{d}(v) = \omega _d*k_{d}(v)$. In total 30 linear bins are split for horizontal axis.

To explain the positive correlation observed both in the degree of a node between two different orders and in the strength of a node between two different orders, we investigated the correlation between the *d*-strength and *d*-degree of a node, in every dataset as shown in Figs. 9 and 10. We find that the *d*-strength of a node is approximately a linear function of the *d*-degree of the node at each order. In particular, we found that, given a node *v*, $s_d(v) \approx \omega _d\ * k_d(v)$, where $\omega _d$ is the average number of activations of a hyperlink of order *d*.

The degree and strength of each node for any order remain the same in a real-world network and its three randomized networks except that the strength of nodes in ${\mathscr {H}}^2_d$ differs from that in the other networks. In ${\mathscr {H}}^2_d$, $s_d(v) = \omega _d\ * k_d(v)$ is expected for each order *d* and confirmed in Figs. S13 and S14 (in Supplementary Material), since the time series of order *d* hyperlinks are swapped in ${\mathscr {H}}^2_d$. This linear function $s_d(v) = \omega _d\ * k_d(v)$ observed in each real-world network approximately, means that the average number of times a node interacts with an order *d* group (the ratio of the *d*-strength to the *d*-degree of the node) is a constant, independent of the number of distinct order *d* groups the node interacts with. Thus, engaging in more groups of a given order *d* will not affect an individual’s average number of interactions per group. The positive correlation in the degree of a node between two different orders, together with the linear relation found between the *d*-strength and *d*-degree of a node, explains the positive correlation found in the strength of a node between two different orders.

### Temporal correlation of events at a local egonetwork

**Figure 11:**
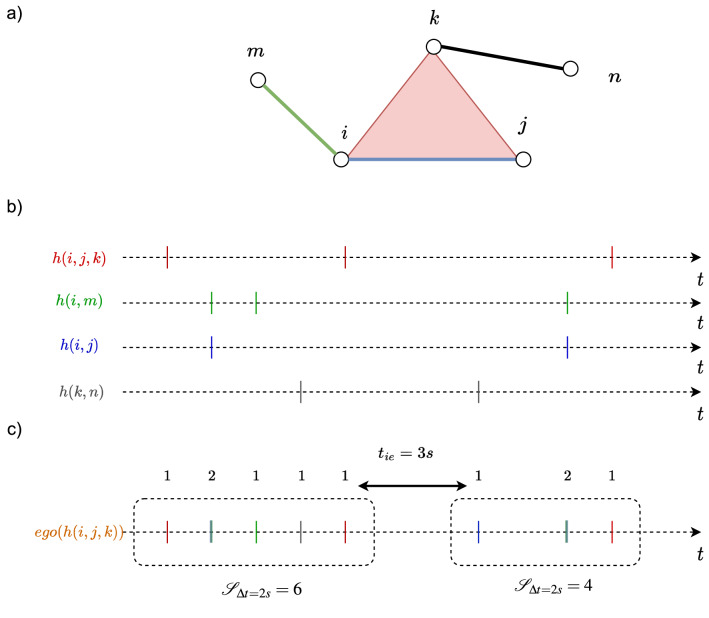
Schematic representation of (**a**) the egonetwork of the hyperlink *h*(*i*, *j*, *k*) , i.e. *ego*(*h*(*i*, *j*, *k*)), (**b**) the time series associated to links belonging to *ego*(*h*(*i*, *j*, *k*)) , (**c**) the time series of the activity of *ego*(*h*(*i*, *j*, *k*)) , which is the sum of the time series of hyperlinks belonging to the egonetwork, and its event trains identified when $\Delta t = 2s$.

Since higher-order events overlap in topology, e.g., the component nodes of a higher-order event tend to participate in events of a lower order, we explore further the temporal correlation of events that occur locally in topology. The topological neighborhood of a hyperlink $h_d$ of order *d*, so called the egonetwork $ego(h_d)$ centered at $h_d$, is defined as the union of the hyperlink $h_d$ and all hyperlinks with an order lower than *d* that share at least one node with $h_d$ in the higher-order aggregated network. We construct the time series of the aggregated activity of an egonetwork $ego(h_d)$, as the sum of the time series of hyperlinks belonging to $ego(h_d)$, as shown in Fig. 11. We then evaluate the temporal correlation of the time series of an egonetwork $ego(h_d)$, to understand whether the activation of the center hyperlink $h_d$ tend to cluster in time with the activation of the other low order hyperlinks in the egonetwork $ego(h_d)$

.

Our analysis method is based on the concept of event trains, proposed by Karsai et al.^5^. A train of events is a sequence of consecutive events whose inter-event times are shorter than or equal to a reference temporal interval $\Delta t$ and separated from the other contacts by an inter-event times larger than $\Delta t$. Given a $\Delta t$ and an activity time series of an egonetwork $ego(h_d)$, trains can be identified, as exemplified in Fig. 11. Given $\Delta t$ and an order *d*, we identify all the trains for each activity series of the egonetwork centered at each order *d* hyperlink. The size of a train is the number of events the train contains. Then, we examine the size distribution $Pr[{\mathscr {S}}^*_{d} = s]$ of the identified trains in which a center hyperlink has been activated at least once. The timescales of physical contacts and collaboration networks are different. The two classes are measured per step of seconds and day respectively. To illustrate our method and findings we consider $\Delta t = 60 s$ (60*d*) in physical contact (collaboration) networks to identify the trains in each ego network. The choice $\Delta t = 60 s$ is also motivated by the observation in Fig. 1 that we start to observe the positive temporal and topological correlation of higher-order events since $\Delta t$ is about 100*s* in physical contact networks. Moreover, we observe the same when $\Delta t = 120 s$ (120*d*) in physical contact (collaboration) networks in the coming analysis.

**Figure 12:**
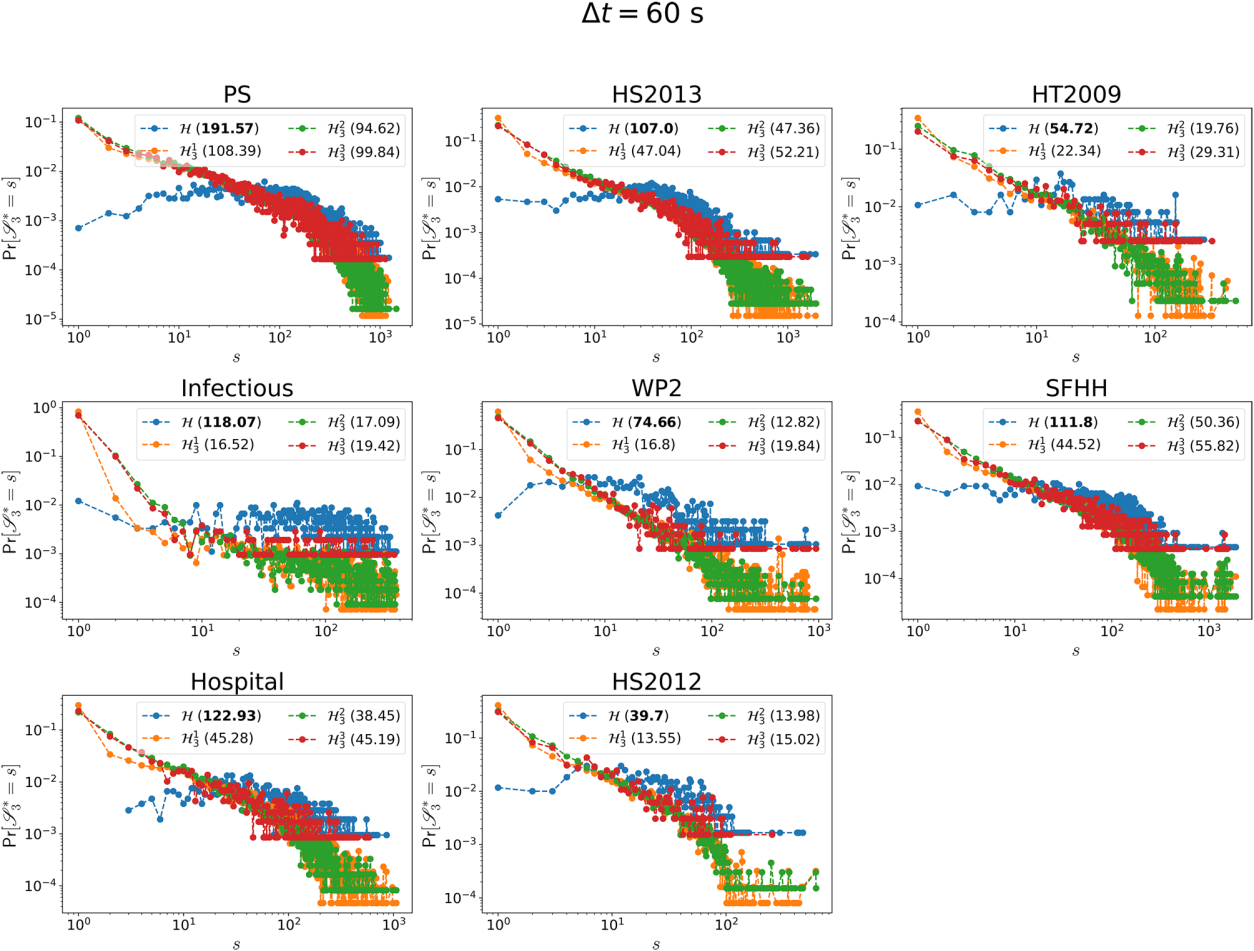
Probability distribution $Pr[{\mathscr {S}}^*_{3}=s]$ of the size ${\mathscr {S}}^*_{3}$ of trains (obtained from the activity series of egonetworks centered at each order 3 hyperlink), where a center link is activated at least once, in each physical contact network ${\mathscr {H}}$ (blue) and its three randomized reference models ${\mathscr {H}}^1_3$ (yellow), ${\mathscr {H}}^2_3$ (green) and ${\mathscr {H}}^3_3$ (red). To identify the trains, we consider $\Delta t = 60s$. For each network, the average size of the trains is reported. The maximum average size among network ${\mathscr {H}}$, ${\mathscr {H}}^1_3$, ${\mathscr {H}}^2_3$ and ${\mathscr {H}}^3_3$ is in bold. The horizontal and vertical axes are presented in logarithmic scale.

**Figure 13:**
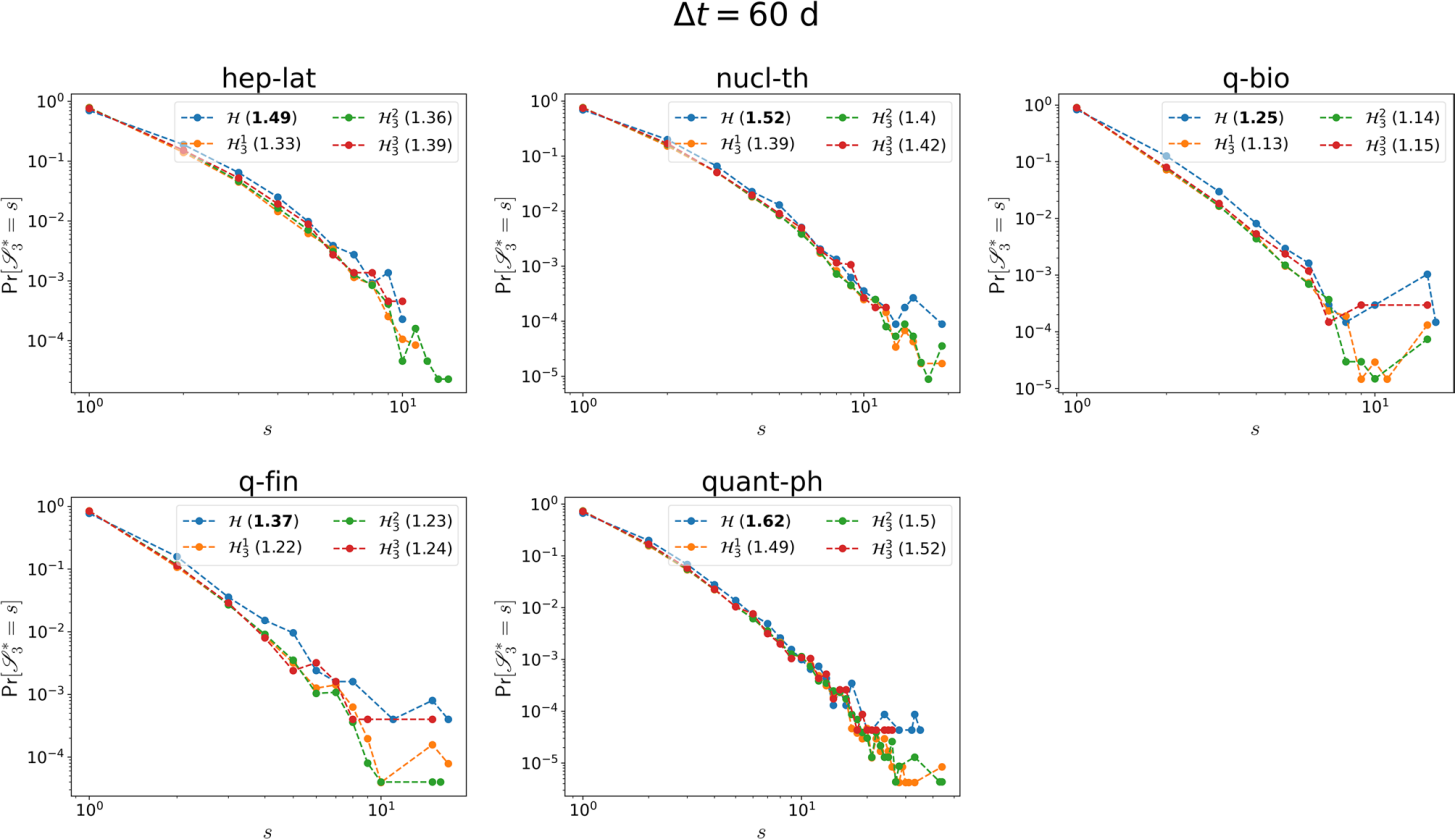
Probability distribution $Pr[{\mathscr {S}}^*_{3}=s]$ of the size ${\mathscr {S}}^*_{3}$ of trains (obtained from the activity series of egonetworks centered at each order 3 hyperlink), where a center link is activated at least once, in each collaboration network ${\mathscr {H}}$ (blue) and its three randomized reference models ${\mathscr {H}}^1_3$ (yellow), ${\mathscr {H}}^2_3$ (green) and ${\mathscr {H}}^3_3$ (red). To identify the trains, we consider $\Delta t = 60s$. For each network, the average size of the trains is reported. The maximum average size among network ${\mathscr {H}}$, ${\mathscr {H}}^1_3$, ${\mathscr {H}}^2_3$ and ${\mathscr {H}}^3_3$ is in bold. The horizontal and vertical axes are presented in logarithmic scale.

Figures 12 and 13 show the train size distribution $Pr[{\mathscr {S}}^*_{3} = s]$ of the egonetworks centered at each order 3 hyperlink in each physical and collaboration network ${\mathscr {H}}$ and its three null models ${\mathscr {H}}^1_{3}$, ${\mathscr {H}}^2_{3}$, ${\mathscr {H}}^3_{3}$. Only order 3 events have been randomized in the three randomized reference models ${\mathscr {H}}^1_{3}$, ${\mathscr {H}}^2_{3}$, and ${\mathscr {H}}^3_{3}$ while the set of events of any other order $d'\ne 3$ remain unchanged in each real-world network and its corresponding randomized network ${\mathscr {H}}_{3}^1$, ${\mathscr {H}}_{3}^2$, ${\mathscr {H}}_{3}^3$. In physical contact networks, the train size is evidently larger on average than that in their corresponding randomized networks. This indicates that an order 3 event tend to occur close in time with many local order 2 events, forming large trains. The trains in collaboration networks are, however, not evidently longer than those in randomized reference models on average. We found similar when considering $\Delta t = 120s$ for physical contacts and $\Delta t = 120d$ for collaboration networks (see Figs. S15 and S16 in Supplementary Material).

The temporal correlation analysis of local events helps explain the interrelation of topological and temporal distance of higher-order events discovered in “Correlation of temporal and topological distance of events” subsection. In physical contact (collaboration) networks, we observe evident (no evident) correlation between topological and temporal distance of events with different orders. Consistently, whereas events overlap in component nodes in both types of networks, local events, thus events close in topology are strongly (weakly or not) correlated in time, in forming long trains, in physical contact (collaboration) networks. In networks where the interrelation between topological and temporal distance of events is more evident (e.g., Infectious and WP2), the correlation of local events in time also tends to be stronger (average train size observed in real-work network is evidently larger than that of randomized reference models). We observe similar results also for the distribution $Pr[{\mathscr {S}}^{*}_ 4 = s]$ of the size ${\mathscr {S}}^{*}_ 4$ of trains obtained from the activity series of ego networks centered at each order 4 hyperlink, as shown in Figs. S17, S18, S19 and S20 in Supplementary Material.

The detected differences between physical contact and collaboration networks may be explained by the fact that physical interactions are driven by physical proximity. For example, individuals that have a group interaction are close in physical distance, which may facility the interaction of a subgroup, resulting in events close in time and topology.

Finally, we discuss briefly whether our finding of the temporal-topological correlation in higher-order temporal networks is still valid taking into account that the higher-order temporal networks we constructed is likely imprecise. The physical contact networks measured are possibly incomplete, influencing the resultant higher-order temporal networks. If the $$\left( {\begin{array}{c}d\\ 2\end{array}}\right)$$ pair-wise contacts of an order *d* event are not observed completely but with one contact missing, the observed higher-order network would be composed of two order $d-1$ events. Hence, we will add such potential missing contacts back to our pair-wise physical contact networks, re-construct the corresponding higher-order networks and explore whether similar temporal-topological correlation could be still be observed. We examine each pair-wise physical contact network at each time step, identify all subgraphs that are composed of a clique of size $d>3$ with one missing link, add such missing links to original pair-wise physical contact networks and construct the corresponding higher-order networks ${\mathscr {H}}_{miss}$ as described in “Datasets” section. Figure S21 (in Supplementary Material) shows the slight change in the number of events of each order in ${\mathscr {H}}_{miss}$ symbol where the missing links have been added. The general observation of the temporal-topological correlation and Infectious and WP2 being among the networks with the strongest correlation holds also for ${\mathscr {H}}_{miss}$ , as shown in Figs. S22 and S23 (Supplementary Material) for order $d=3$ and $d=4$, respectively.

## Conclusion

In this paper, we have proposed a method to systematically characterize temporal and topological properties of events of arbitrary orders. We applied our methods to 8 physical contact and 5 collaboration higher-order evolving networks and observe their difference. In physical contacts, events relatively close in time tend to occur also close in topology. Moreover, events usually overlap in component nodes and these local events overlapping in component nodes are also usually correlated in time. Such temporal correlation of local events supports again the correlation between temporal and topological distances of events observed in our first analysis. Differently, in collaboration networks, the temporal and topological correlation of events is either weak or absent. Despite events also overlap in component nodes, their temporal correlation almost disappears in collaboration networks. The detected dissimilarities between physical contacts and collaboration networks could be related to a fundamental difference between the two kind of networks. In physical contacts individuals participate in events driven by physical proximity. The physical proximity of individuals that participate in a higher-order event may facilitate interaction of them or a subgroup in the near future. The time of scientific collaborations are likely driven more by their content and creation process.

Via our analysis of the topological overlap of events with different orders in component nodes, we also observe similarities between the two kinds of networks. Nodes that participate in many events (groups) of a given order tend to interact in many events (groups) of a different order. Hence, nodes are consistent in interactions with respect to frequency and diversity across different orders.

Our method explores the temporal and topological relation of the basic building block of events, the activations of fully connected cliques. A promising direction could be generalizing this method to the activations of relevant motifs, and to investigate the interplay between topological location and temporal delay of such structures. Beyond, our method can be applied to compare different classes of networks (e.g. biological, brain or collaboration networks) and to explore how detected properties/patterns of a network can influence the dynamic processes unfolding on the network. Finally, the topological and temporal properties of events detected in this paper could foster higher-order evolving network models that better reproduce patterns observed so far.

## Supplementary Information

Supplementary Figures.

## Data Availability

We are pleased to make available the source-code and datasets accompanying this research. The SocioPatterns data are available at http://www.sociopatterns.org the analyzed arxiv dataset (updated until 29-10-2021) at https://surfdrive.surf.nl/files/index.php/s/L0UluLjtf7iHkGp. Last update of arxiv dataset is publicly available at https://www.kaggle.com/datasets/Cornell-University/arxiv.
