# Supplementary material for: Temporal-topological properties of higher-order evolving networks
Source: Sci Rep. 2023 Apr 11;13:5885. doi: 10.1038/s41598-023-32253-9 (PMC10090145; doi:10.1038/s41598-023-32253-9)
Supplement: Supplementary file 1 — Supplementary Figures. [file 41598_2023_32253_MOESM1_ESM.pdf]

Temporal-topological properties of higher-order evolving networks
Supplementary Material

Alberto Ceria[1,*] and Huijuan Wang[1]

[1]Faculty of Electrical Engineering, Mathematics, and Computer Science, Delft University of Technology, Mekelweg 4, 2628 CD, Delft, The Netherlands
[*]A.Ceria@tudelft.nl

A General Statistics

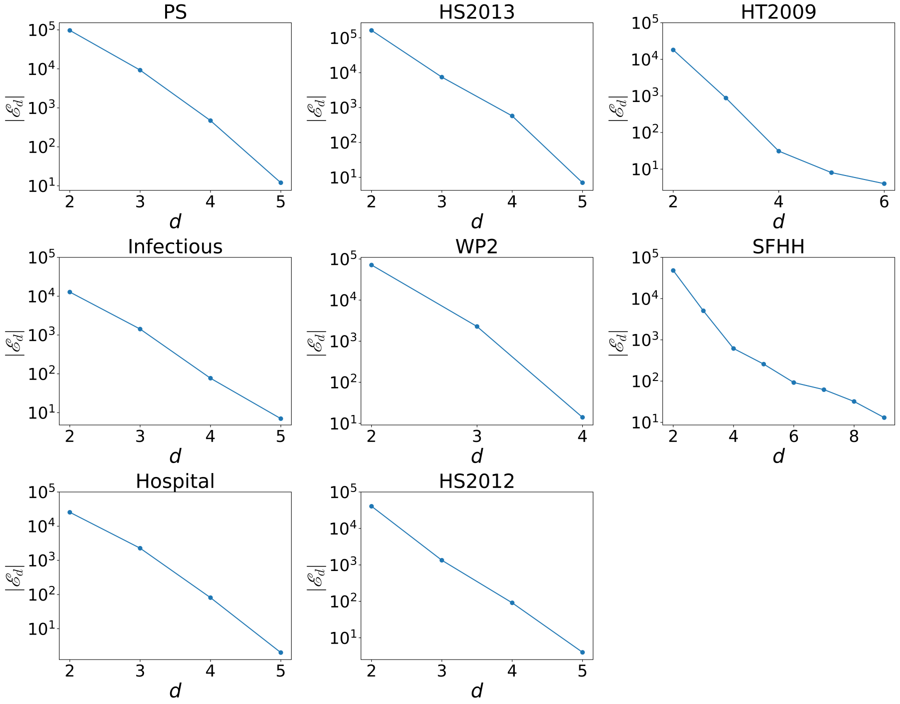

Figure S1. Total number of events ($|\mathscr{E}_d|$) for each order d in physical contact networks. Vertical axis is presented in logarithmic scale.

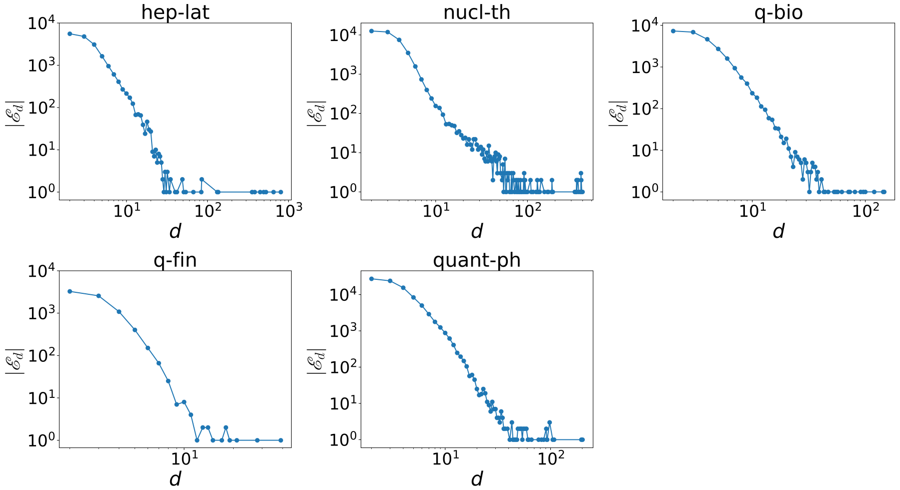

Figure S2. Total number of events ($|\mathscr{E}_d|$) for each order d in collaboration networks. Vertical and horizontal axes are presented in logarithmic scale.

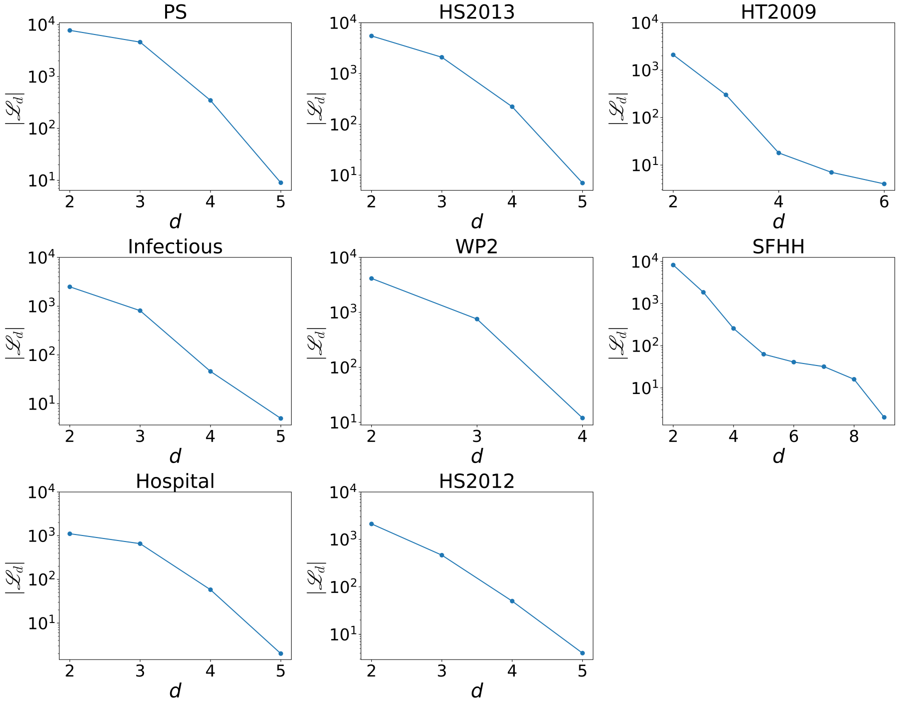

Figure S3. Total number of hyperlinks ($|\mathscr{L}_d|$) in the time aggregated higher-order network for each order d for physical contact datasets. Vertical axis is presented in logarithmic scale.

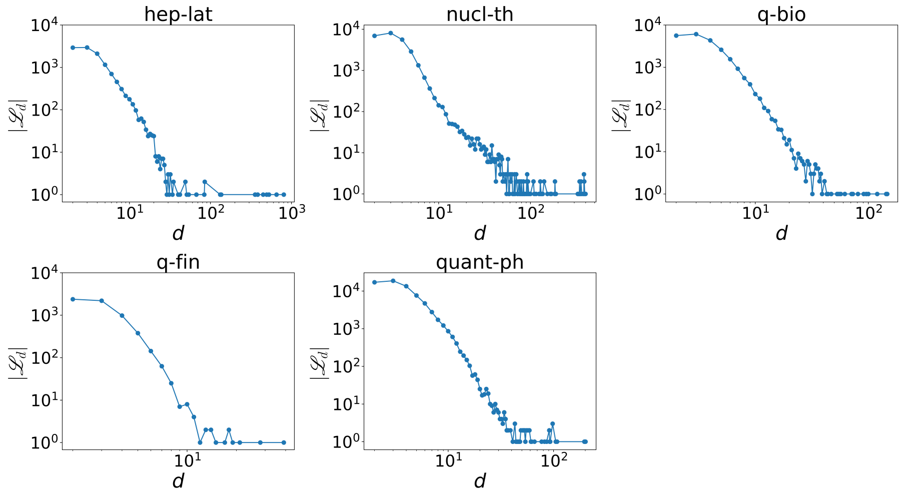

Figure S4. Total number of hyperlinks ($|\mathscr{L}_d|$) for each order d in collaboration networks. Vertical and horizantal axes are presented in logarithmic scale.

B Temporal-topological correlation of events

B.1 Correlation of temporal and topological distance of events

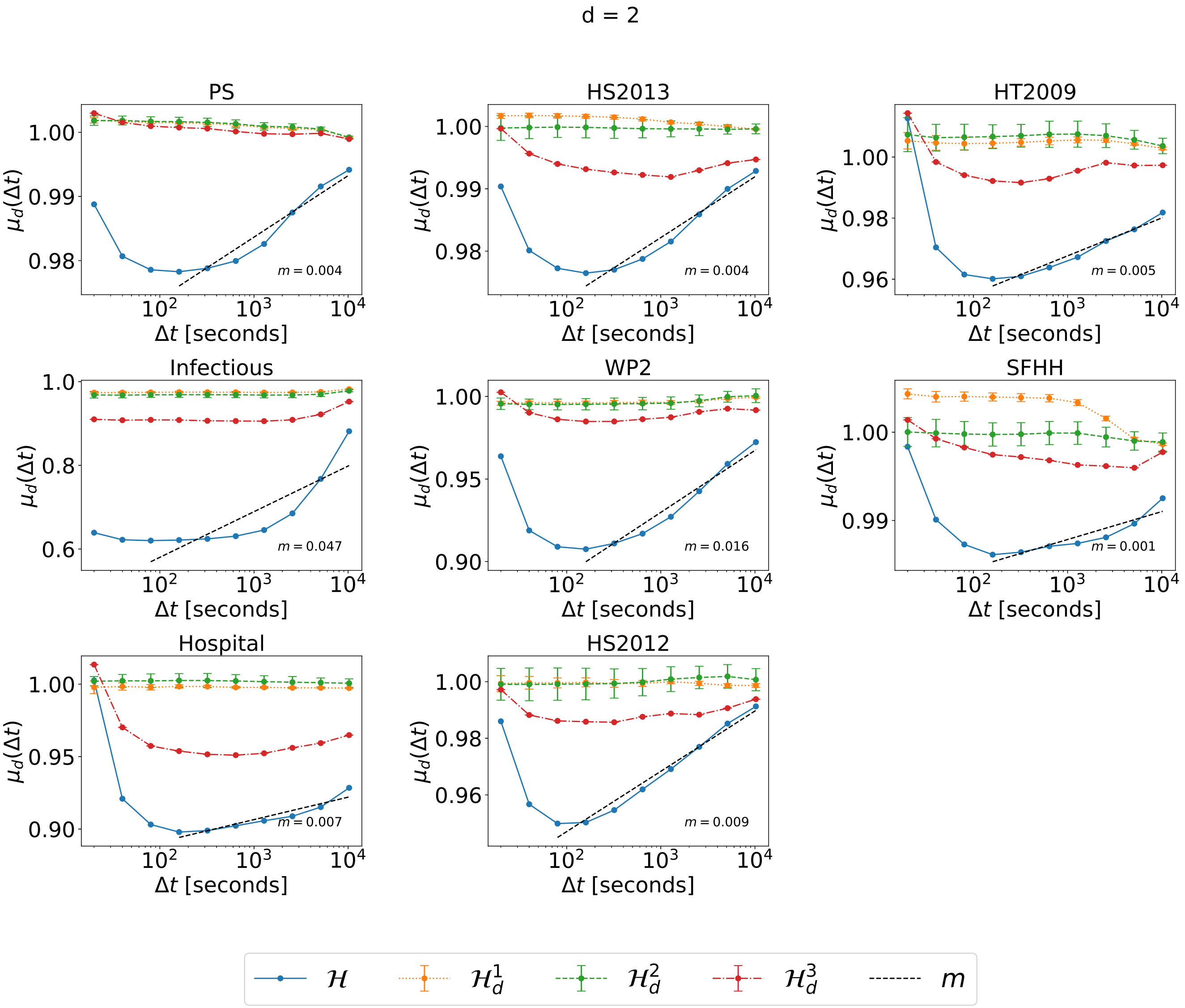

Figure S5. The normalized average topological distance $\mu_d(\Delta t) = \frac{E[\eta(e,e')|\mathscr{T}(e,e')<\Delta t,\ e\in\mathscr{E}_d,\ e'\in\mathscr{E}\setminus\mathscr{E}_d]}{E[\eta(e,e')|\ e\in\mathscr{E}_d,\ e'\in\mathscr{E}\setminus\mathscr{E}_d]}$, between an order $d = 2$ event and an event of a different order, in each physical contact network and its corresponding three randomized null models $\mathscr{H}_d^1$ (yellow), $\mathscr{H}_d^2$ (green) and $\mathscr{H}_d^3$ (red), which preserve or destroy specific properties of order $d = 3$ events. $\lim_{\Delta t\to\infty} E[\eta(e,e')|\mathscr{T}(e,e') < \Delta t,\ e \in \mathscr{E}_d,\ e' \in \mathscr{E}\setminus\mathscr{E}_d] = E[\eta(e,e')|\ e \in \mathscr{E}_d,\ e' \in \mathscr{E}\setminus\mathscr{E}_d]$ for any d. The horizontal axes are presented in logarithmic scale. The dashed line in each figure corresponds to the linear fit (with slope m) of $\mu_d(\Delta t)$ as a function of $log_{10}(\Delta t)$ in $\mathscr{H}$, for the part that the curve has an increasing trend. For each dataset, the results of the three corresponding randomized models are obtained from 10 independent realizations.

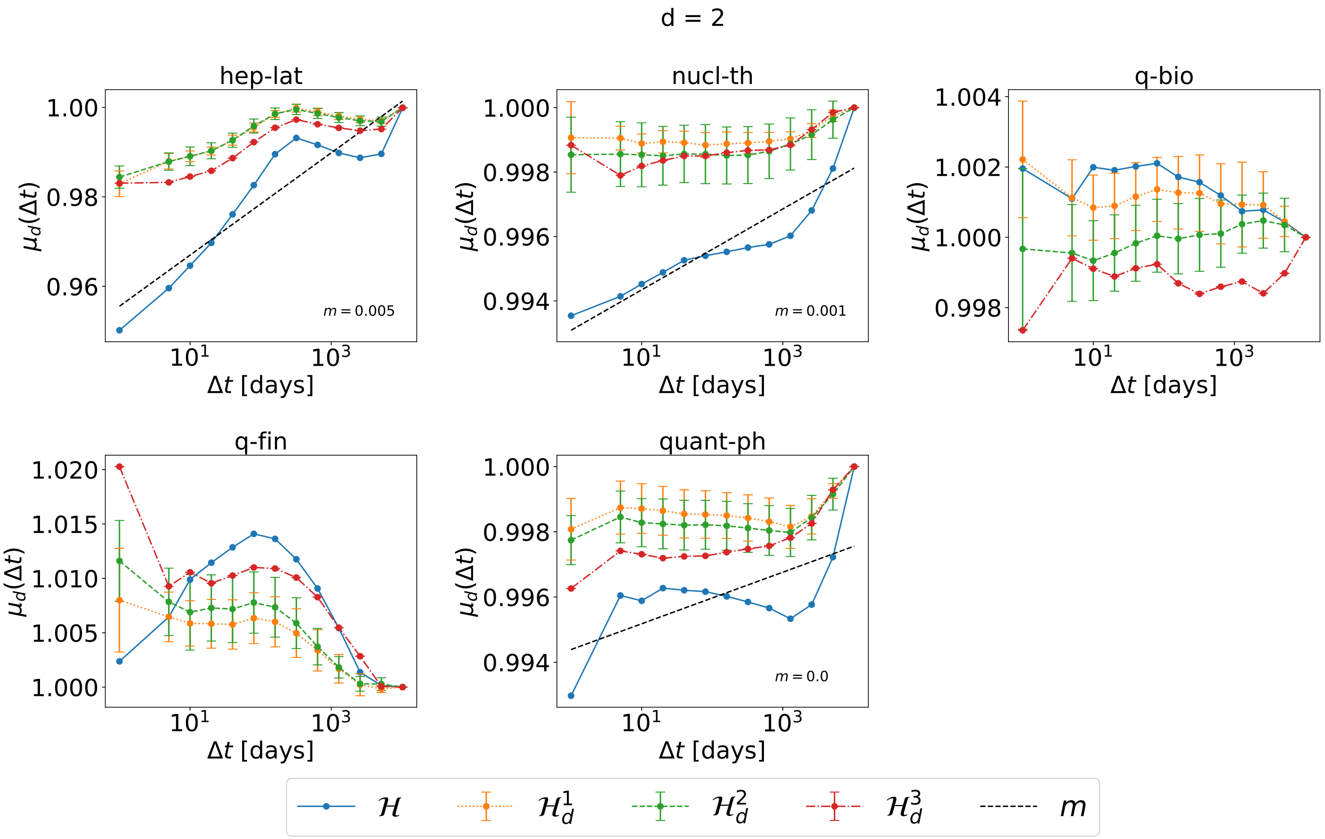

Figure S6. The normalized average topological distance $\mu_d(\Delta t) = \frac{E[\eta(e,e')|\mathscr{T}(e,e')<\Delta t,\ e\in\mathscr{E}_d,\ e'\in\mathscr{E}\setminus\mathscr{E}_d]}{E[\eta(e,e')|\ e\in\mathscr{E}_d,\ e'\in\mathscr{E}\setminus\mathscr{E}_d]}$, between an order $d = 2$ event and an event of a different order, in each collaboration network and its corresponding three randomized null models $\mathscr{H}_d^1$ (yellow), $\mathscr{H}_d^2$ (green) and $\mathscr{H}_d^3$ (red), which preserve or destroy specific properties of order $d = 2$ events. $\lim_{\Delta t\to\infty} E[\eta(e,e')|\mathscr{T}(e,e') < \Delta t,\ e \in \mathscr{E}_d,\ e' \in \mathscr{E} \setminus \mathscr{E}_d] = E[\eta(e,e')|\ e \in \mathscr{E}_d,\ e' \in \mathscr{E} \setminus \mathscr{E}_d]$ for any d. The horizontal axes are presented in logarithmic scale. The dashed line in each figure corresponds to the linear fit (with slope m) of $\mu_d(\Delta t)$ as a function of $log_{10}(\Delta t)$ in $\mathscr{H}$, for the part that the curve has an increasing trend. For each dataset, the results of the three corresponding randomized models are obtained from 10 independent realizations.

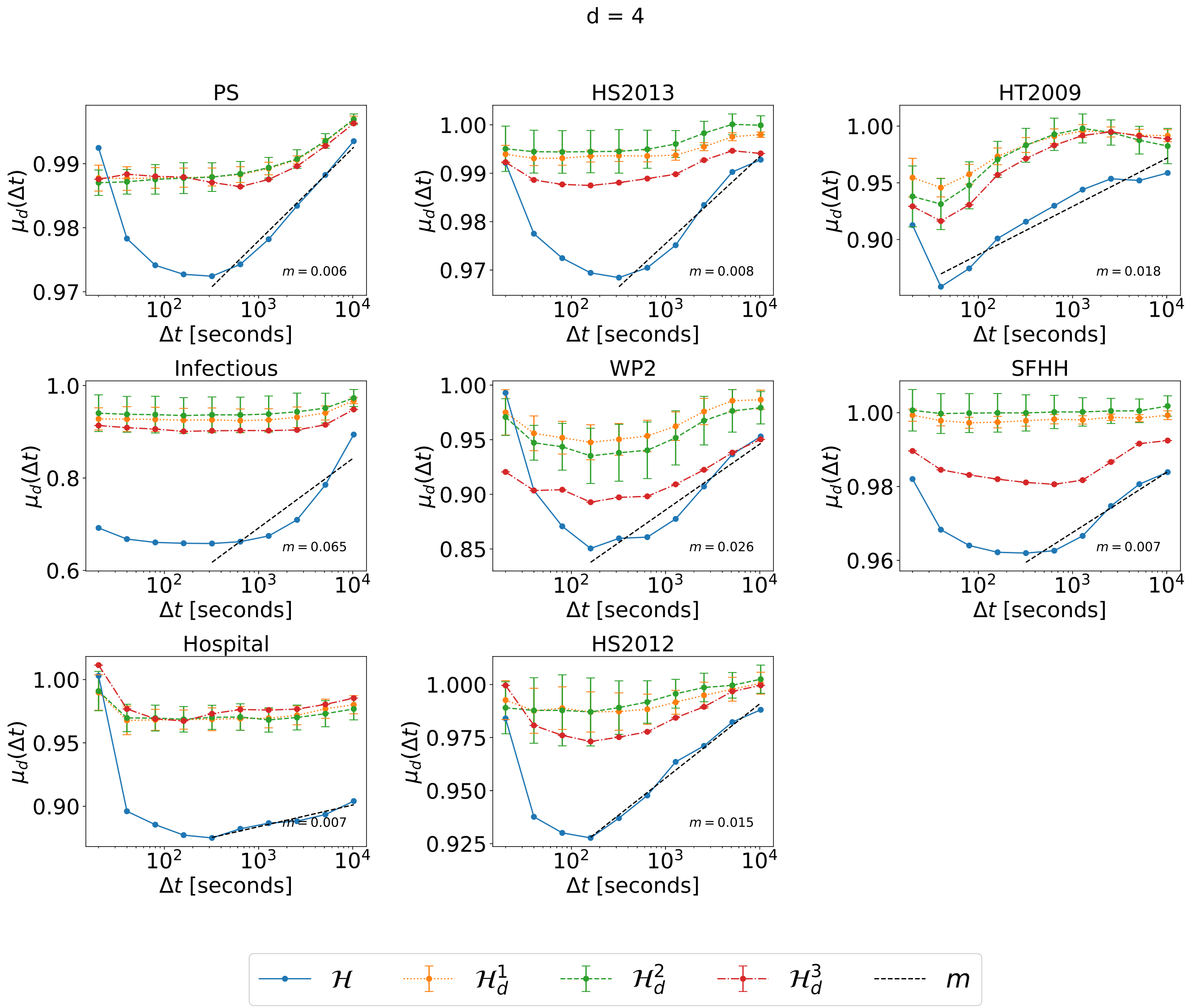

Figure S7. The normalized average topological distance $\mu_d(\Delta t) = \frac{E[\eta(e,e')|\mathscr{T}(e,e')<\Delta t,\ e\in\mathscr{E}_d,\ e'\in\mathscr{E}\setminus\mathscr{E}_d]}{E[\eta(e,e')|\ e\in\mathscr{E}_d,\ e'\in\mathscr{E}\setminus\mathscr{E}_d]}$, between an order $d=4$ event and an event of a different order, in each physical contact network and its corresponding three randomized null models $\mathscr{H}_d^1$ (yellow), $\mathscr{H}_d^2$ (green) and $\mathscr{H}_d^3$ (red), which preserve or destroy specific properties of order $d=4$ events. $\lim_{\Delta t\to\infty} E[\eta(e,e')|\mathscr{T}(e,e')<\Delta t,\ e\in\mathscr{E}_d,\ e'\in\mathscr{E}\setminus\mathscr{E}_d] = E[\eta(e,e')|\ e\in\mathscr{E}_d,\ e'\in\mathscr{E}\setminus\mathscr{E}_d]$ for any d. The horizontal axes are presented in logarithmic scale. The dashed line in each figure corresponds to the linear fit (with slope m) of $\mu_d(\Delta t)$ as a function of $log_{10}(\Delta t)$ in $\mathscr{H}$, for the part that the curve has an increasing trend. For each dataset, the results of the three corresponding randomized models are obtained from 10 independent realizations.

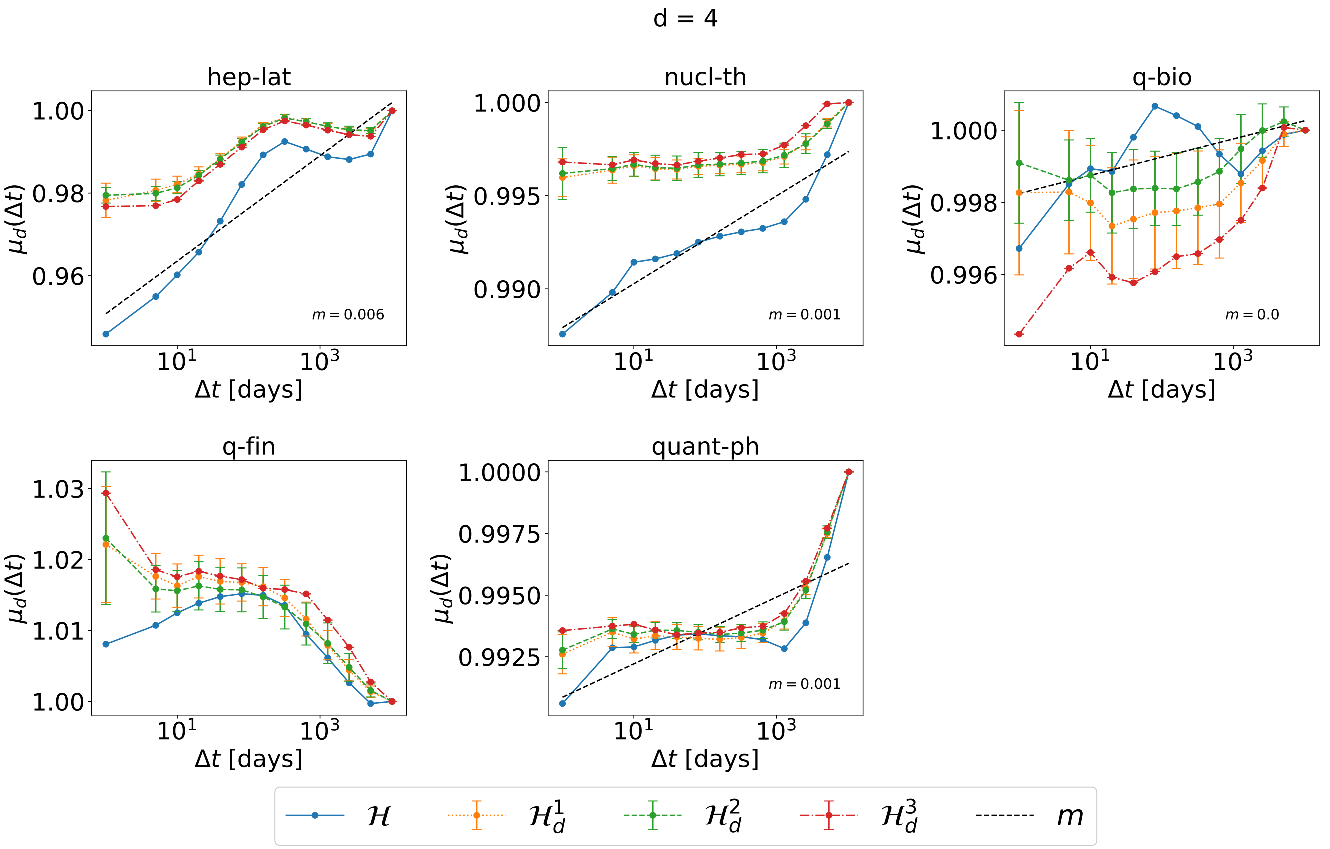

Figure S8. The normalized average topological distance $\mu_d(\Delta t) = \frac{E[\eta(e,e')|\mathscr{T}(e,e')<\Delta t,\ e\in\mathscr{E}_d,\ e'\in\mathscr{E}\setminus\mathscr{E}_d]}{E[\eta(e,e')|\ e\in\mathscr{E}_d,\ e'\in\mathscr{E}\setminus\mathscr{E}_d]}$, between an order $d = 4$ event and an event of a different order, in each collaboration network and its corresponding three randomized null models $\mathscr{H}_d^1$ (yellow), $\mathscr{H}_d^2$ (green) and $\mathscr{H}_d^3$ (red), which preserve or destroy specific properties of order $d = 3$ events. $\lim_{\Delta t \to \infty} E[\eta(e,e')|\mathscr{T}(e,e') < \Delta t,\ e \in \mathscr{E}_d,\ e' \in \mathscr{E} \setminus \mathscr{E}_d] = E[\eta(e,e')|\ e \in \mathscr{E}_d,\ e' \in \mathscr{E} \setminus \mathscr{E}_d]$ for any d. The horizontal axes are presented in logarithmic scale. The dashed line in each figure corresponds to the linear fit (with slope m) of $\mu_d(\Delta t)$ as a function of $log_{10}(\Delta t)$ in $\mathscr{H}$, for the part that the curve has an increasing trend. For each dataset, the results of the three corresponding randomized models are obtained from 10 independent realizations.

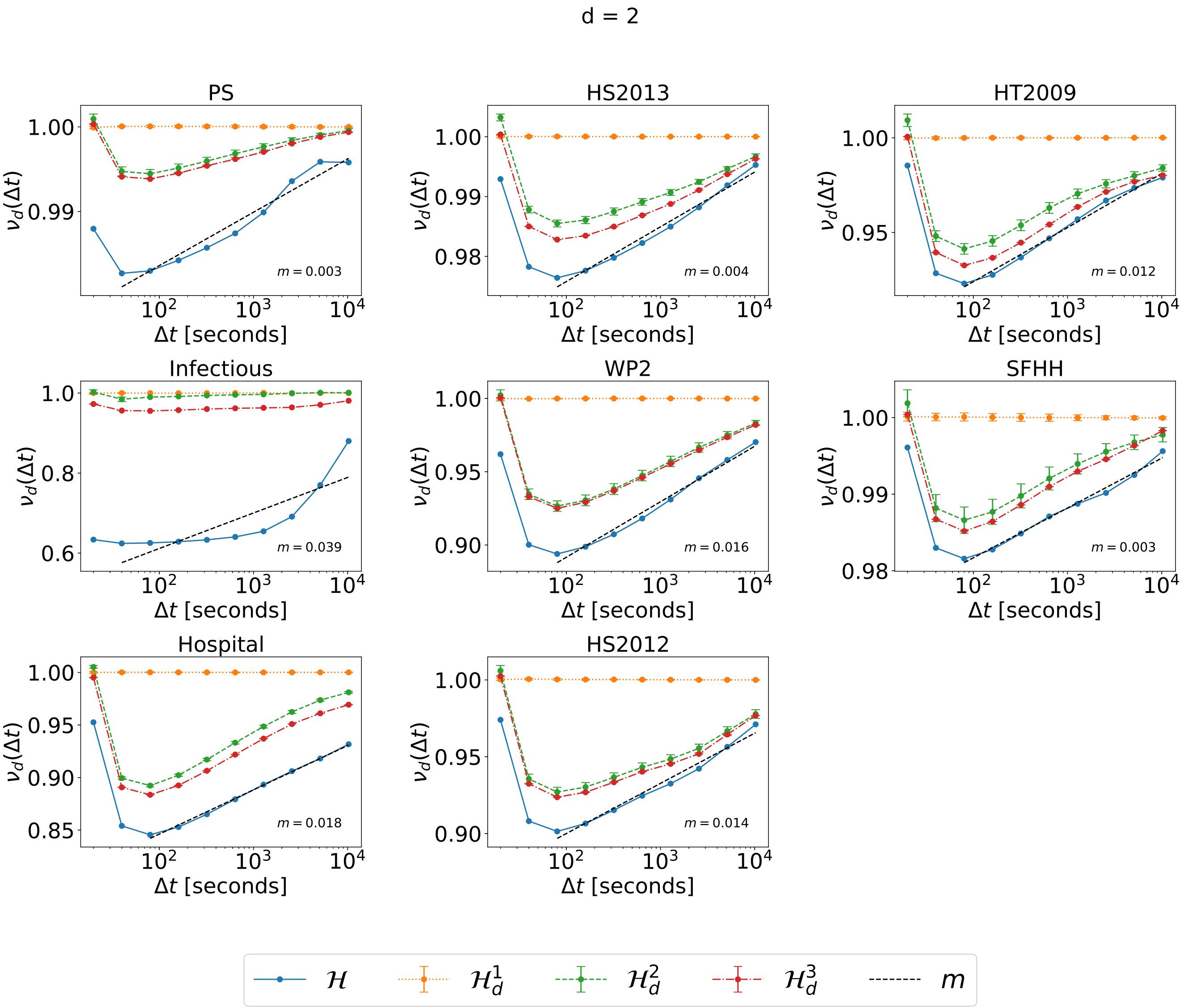

Figure S9. The normalized average topological distance $\nu_d(\Delta t) = \frac{E[\eta(e,e')|\mathscr{T}(e,e')<\Delta t,\ e,\ e'\in\mathscr{E}_d]}{E[\eta(e,e')|\ e,\ e'\in\mathscr{E}_d]}$, between two order $d = 2$ events, in each physical contact network and its corresponding three randomized null models $\mathscr{H}_d^1$ (yellow), $\mathscr{H}_d^2$ (green) and $\mathscr{H}_d^3$ (red), which preserve or destroy specific properties of order $d = 2$ events. $\lim_{\Delta t\to\infty} E[\eta(e,e')|\mathscr{T}(e,e') < \Delta t,\ e,\ e' \in \mathscr{E}_d] = E[\eta(e,e')|\ e,\ e' \in \mathscr{E}_d]$ for any d. The horizontal axes are presented in logarithmic scale. The dashed line in each figure corresponds to the linear fit (with slope m) of $\nu_d(\Delta t)$ as a function of $log_{10}(\Delta t)$ in $\mathscr{H}$, for the part that the curve has an increasing trend. For each dataset, the results of the three corresponding randomized models are obtained from 10 independent realizations.

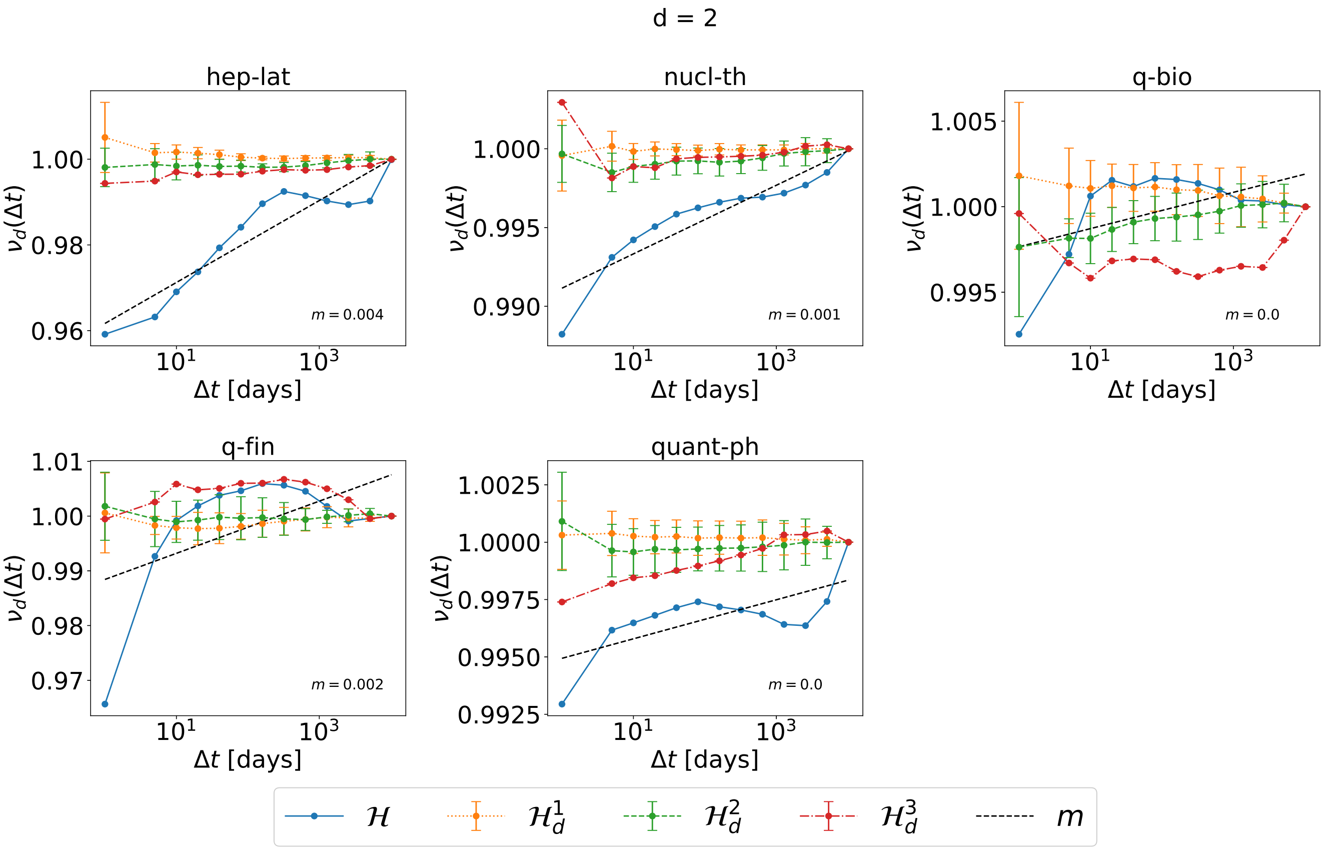

Figure S10. The normalized average topological distance $\nu_d(\Delta t) = \frac{E[\eta(e,e')|\mathscr{T}(e,e')<\Delta t,\ e,\ e' \in \mathscr{E}_d]}{E[\eta(e,e')|\ e,\ e' \in \mathscr{E}_d]}$, between two order $d = 2$ events, in each collaboration network and its corresponding three randomized null models $\mathscr{H}_d^1$ (yellow), $\mathscr{H}_d^2$ (green) and $\mathscr{H}_d^3$ (red), which preserve or destroy specific properties of order $d = 2$ events.
$\lim_{\Delta t \to \infty} E[\eta(e,e')|\mathscr{T}(e,e') < \Delta t,\ e,\ e' \in \mathscr{E}_d] = E[\eta(e,e')|\ e,\ e' \in \mathscr{E}_d]$ for any d. The horizontal axes are presented in logarithmic scale. The dashed line in each figure corresponds to the linear fit (with slope m) of $\nu_d(\Delta t)$ as a function of $log_{10}(\Delta t)$ in $\mathscr{H}$, for the part that the curve has an increasing trend. For each dataset, the results of the three corresponding randomized models are obtained from 10 independent realizations.

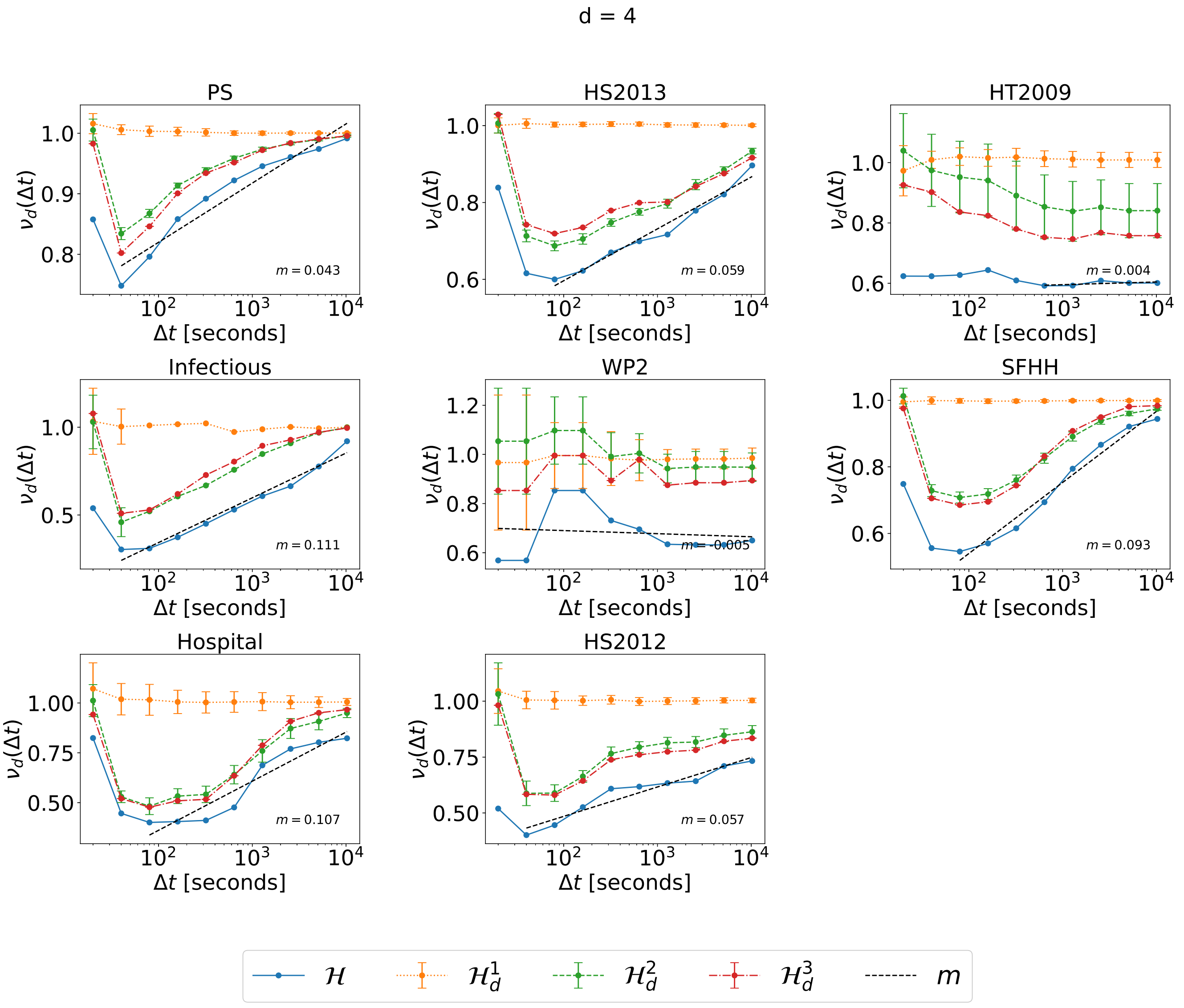

Figure S11. The normalized average topological distance $\nu_d(\Delta t) = \frac{E[\eta(e,e')|\mathcal{T}(e,e')<\Delta t,\ e,\ e' \in \mathcal{E}_d]}{E[\eta(e,e')|\ e,\ e' \in \mathcal{E}_d]}$, between two order $d = 4$ events, in each physical contact network and its corresponding three randomized null models $\mathcal{H}_d^1$ (yellow), $\mathcal{H}_d^2$ (green) and $\mathcal{H}_d^3$ (red), which preserve or destroy specific properties of order $d = 4$ events. $\lim_{\Delta t \to \infty} E[\eta(e,e')|\mathcal{T}(e,e') < \Delta t,\ e,\ e' \in \mathcal{E}_d] = E[\eta(e,e')|\ e,\ e' \in \mathcal{E}_d]$ for any d. The horizontal axes are presented in logarithmic scale. The dashed line in each figure corresponds to the linear fit (with slope m) of $\nu_d(\Delta t)$ as a function of $log_{10}(\Delta t)$ in $\mathcal{H}$, for the part that the curve has an increasing trend. For each dataset, the results of the three corresponding randomized models are obtained from 10 independent realizations.

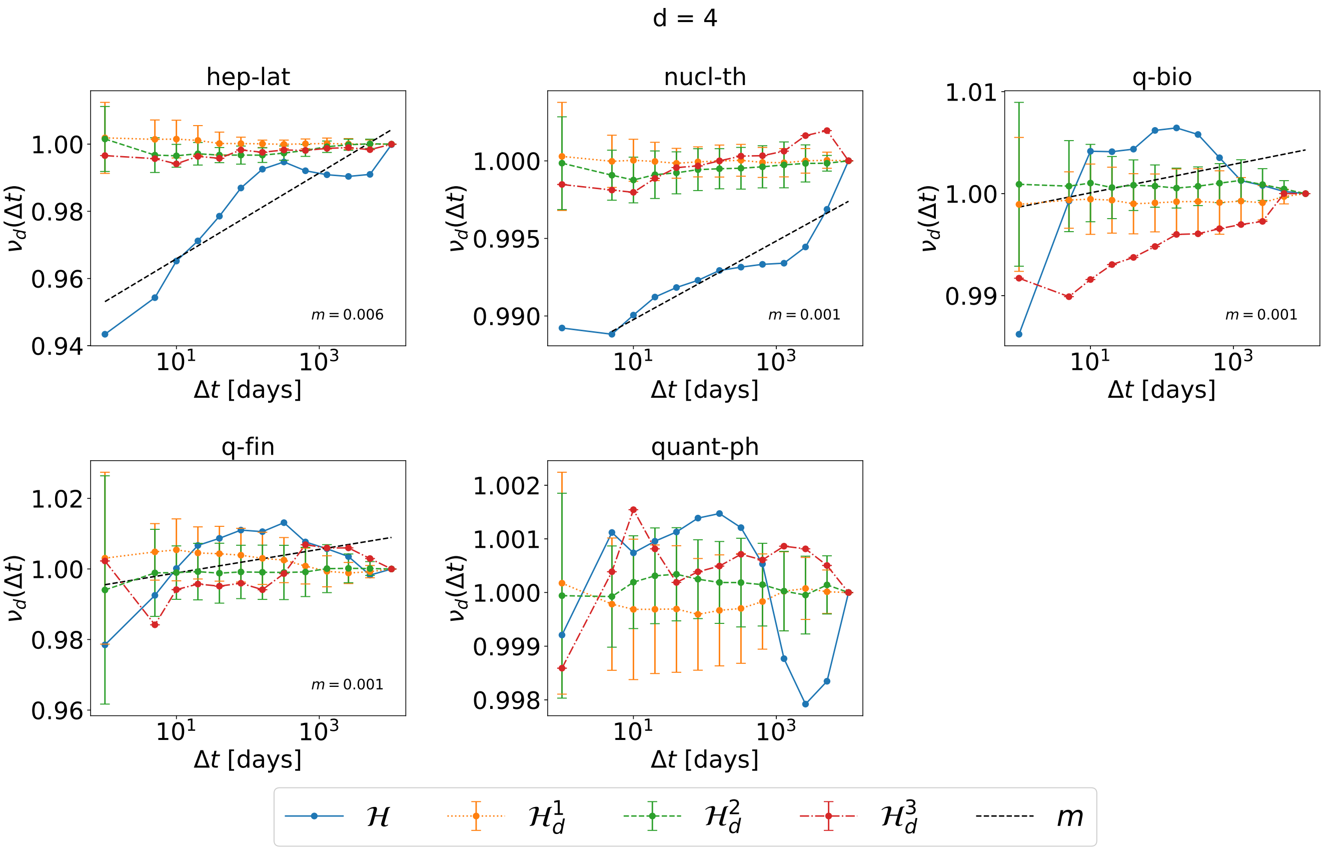

Figure S12. The normalized average topological distance $\nu_d(\Delta t) = \frac{E[\eta(e,e')|\mathscr{T}(e,e')<\Delta t,\ e,\ e' \in \mathscr{E}_d]}{E[\eta(e,e')|\ e,\ e' \in \mathscr{E}_d]}$, between two order $d = 4$ events, in each collaboration network and its corresponding three randomized null models $\mathscr{H}_d^1$ (yellow), $\mathscr{H}_d^2$ (green) and $\mathscr{H}_d^3$ (red), which preserve or destroy specific properties of order $d = 4$ events. $\lim_{\Delta t \to \infty} E[\eta(e,e')|\mathscr{T}(e,e') < \Delta t,\ e,\ e' \in \mathscr{E}_d] = E[\eta(e,e')|\ e,\ e' \in \mathscr{E}_d]$ for any d. The horizontal axes are presented in logarithmic scale. The dashed line in each figure corresponds to the linear fit (with slope m) of $\nu_d(\Delta t)$ as a function of $log_{10}(\Delta t)$ in $\mathscr{H}$, for the part that the curve has an increasing trend. For each dataset, the results of the three corresponding randomized models are obtained from 10 independent realizations.

B.2 Topological correlation of events

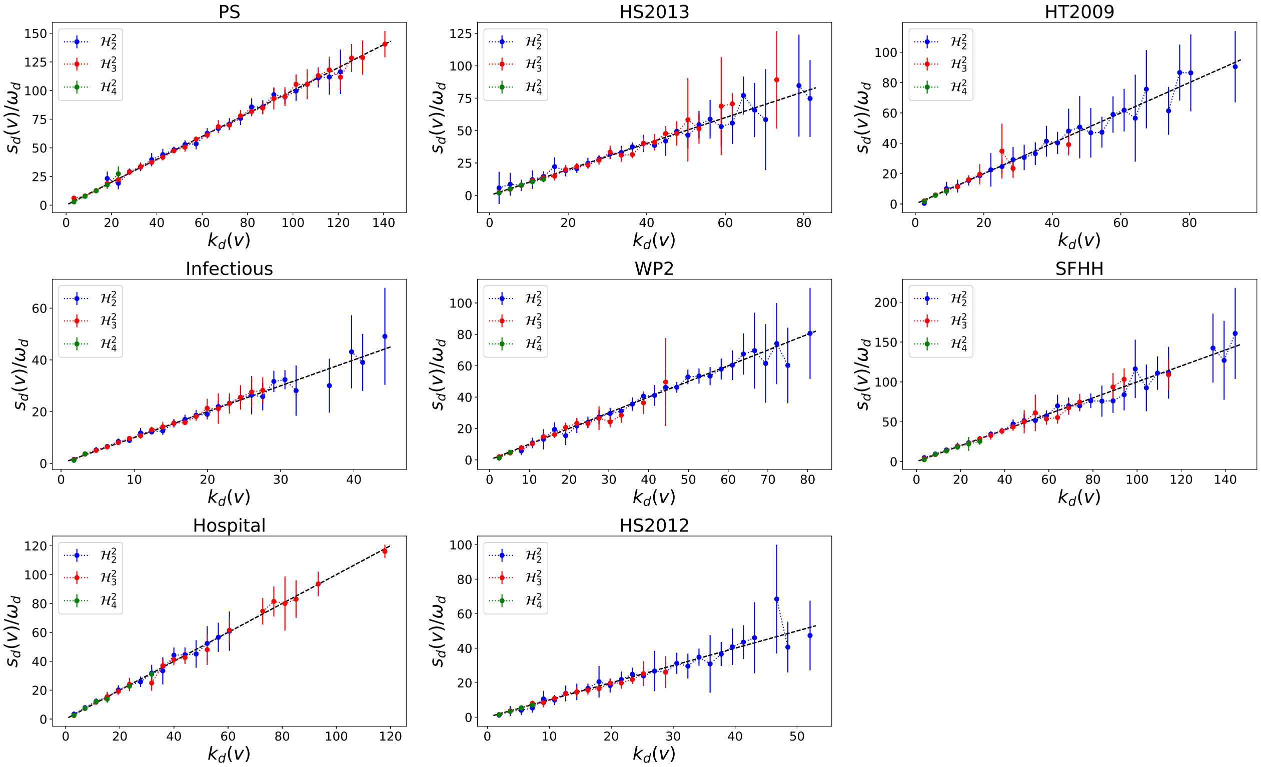

Figure S13. The d-strength $s_d(v)$ versus the the d-degree $k_d(v)$ of a node v in the randomized reference model $\mathscr{H}_d^2$ obtained from each real-world physical contact network, when d is equal to 2 (blue dashed line), 3 (red dashed line) and 4 (green dashed line). The vertical axis is normalized by the average number ω_d of activations of a hyperlink of order d. The black dashed line represents the reference case $s_d(v) = \omega_d * k_d(v)$. The error bar correspond to the standard deviation, centered in the mean value of 10 independent realizations of randomized reference model $\mathscr{H}_d^2$. In total 30 linear bins are split for horizontal axis.

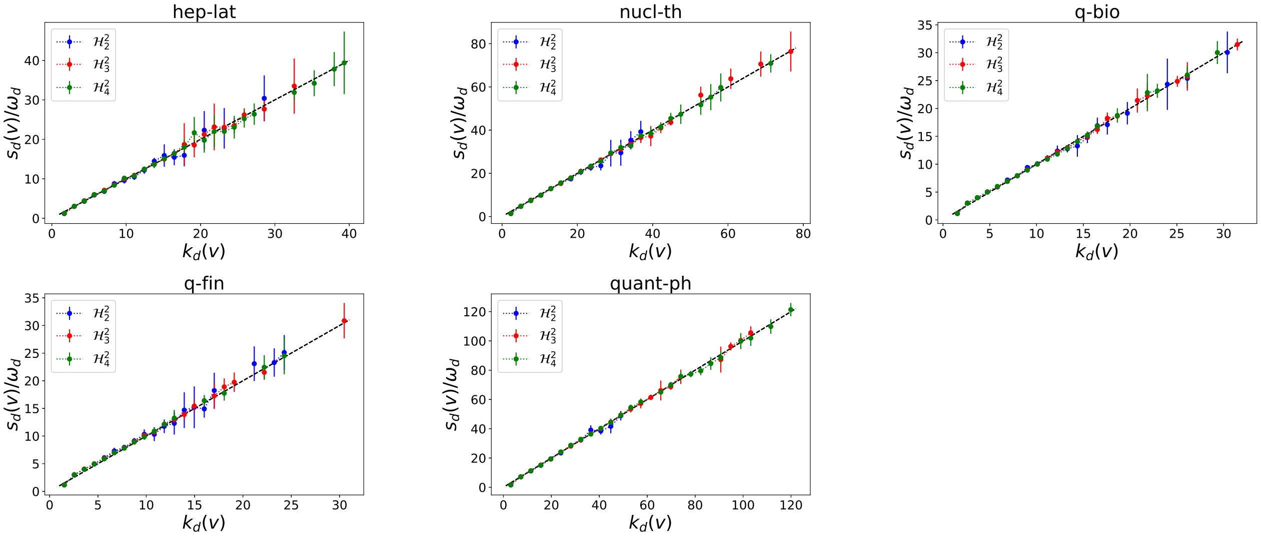

Figure S14. The d-strength $s_d(v)$ versus the the d-degree $k_d(v)$ of a node v of the randomized reference model $\mathscr{H}_d^2$ obtained from each real-world collaboration network, when d is equal to 2 (blue dashed line), 3 (red dashed line) and 4 (green dashed line). The vertical axis is normalized by the average number ω_d of activations of a hyperlink of order d. The black dashed line represents the reference case $s_d(v) = \omega_d * k_d(v)$. The errorbar correspond to the standard deviation, centered in the mean value of 10 independent realizations of randomized reference model $\mathscr{H}_d^2$. In total 30 linear bins are split for horizontal axis.

B.3 Temporal correlation of events at a local ego network

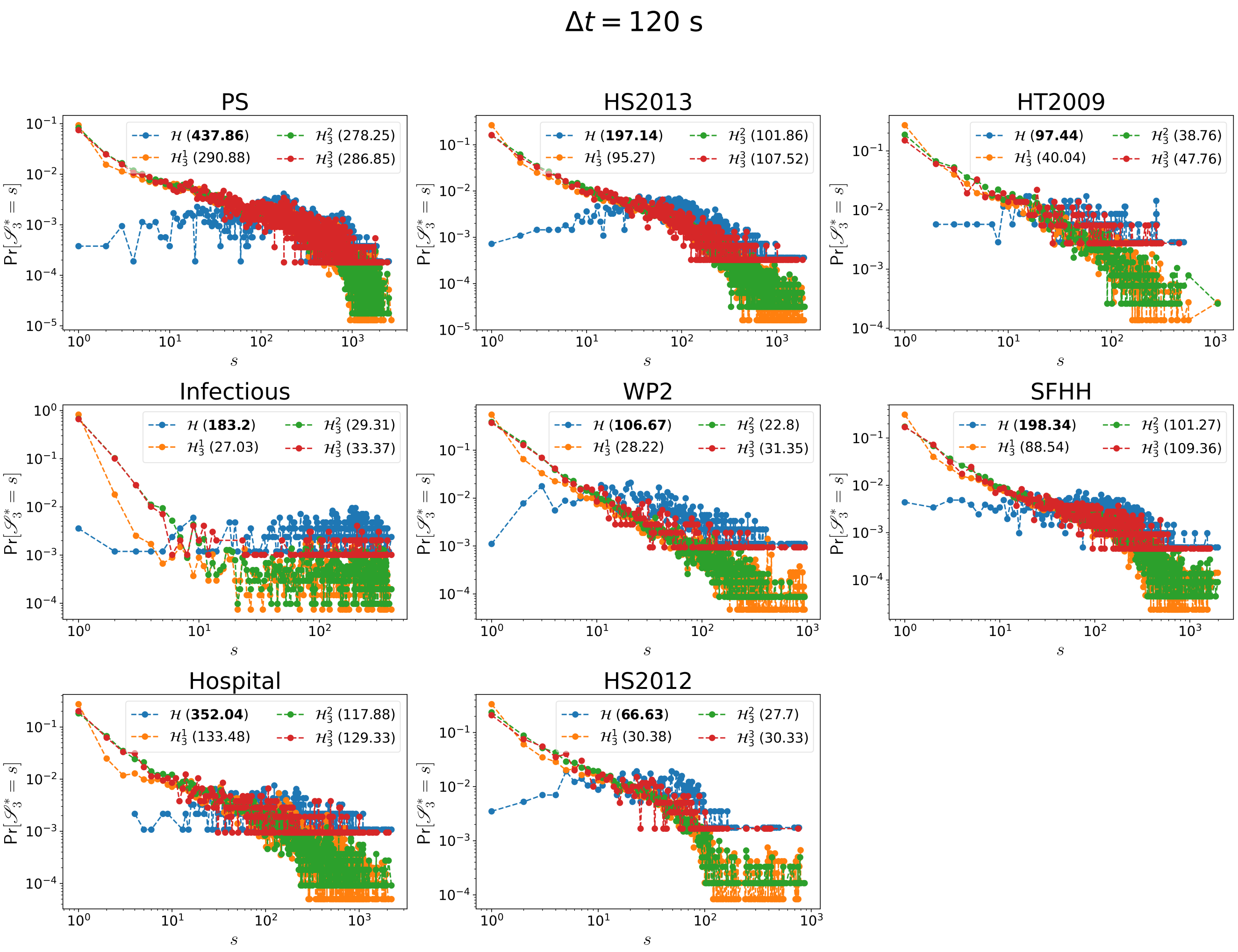

Figure S15. Probability distribution $Pr[\mathscr{S}_3^* = s]$ of the size $\mathscr{S}_3^*$ of trains (obtained from the activity series of egonetworks centered at each order 3 hyperlink), where a center link is activated at least once, in each physical contact network $\mathscr{H}$ (blue) and its three randomized reference models $\mathscr{H}_3^1$ (yellow), $\mathscr{H}_3^2$ (green) and $\mathscr{H}_3^3$ (red). To identify the trains, we consider $\Delta t = 120s$. For each network, the average size of the trains is reported. The maximum average size among network $\mathscr{H}$, $\mathscr{H}_3^1$, $\mathscr{H}_3^2$ and $\mathscr{H}_3^3$ is in bold. The horizontal and vertical axes are presented in logarithmic scale.

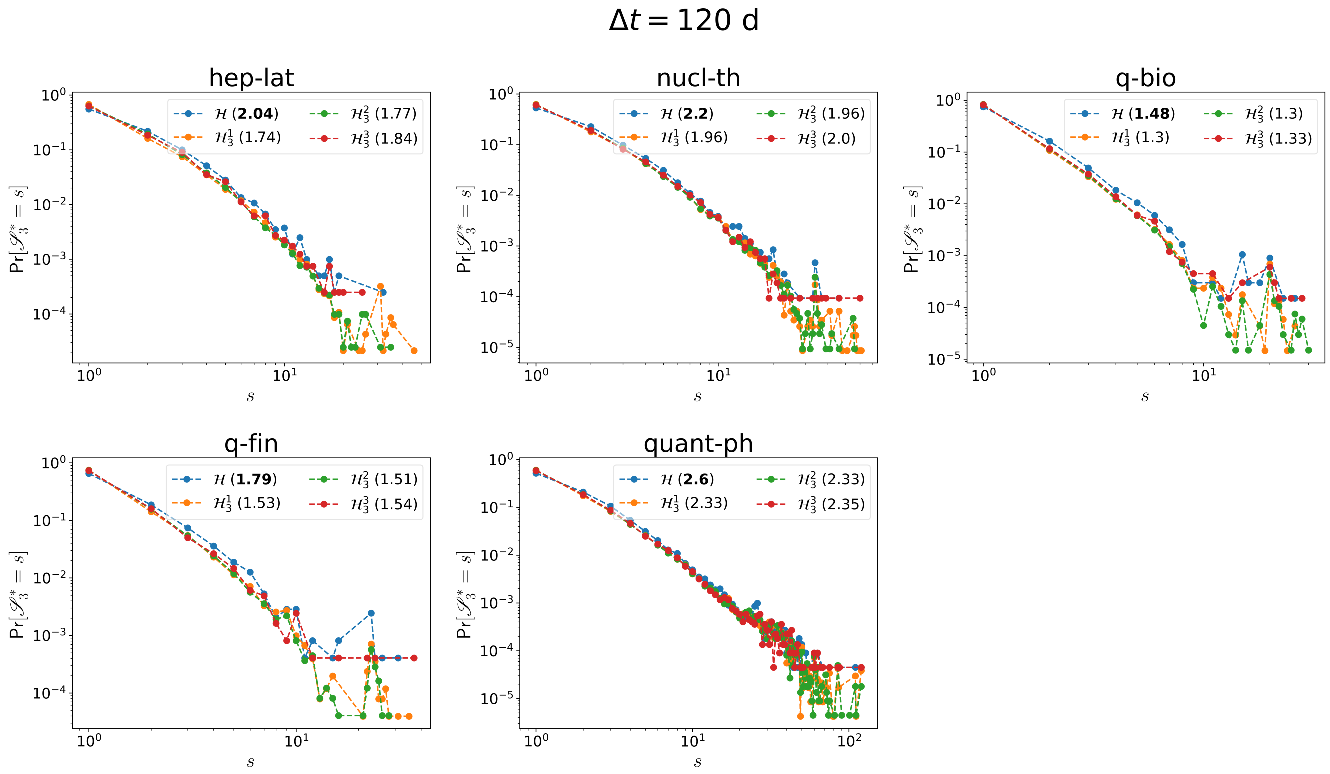

Figure S16. Probability distribution $Pr[\mathscr{S}_3^* = s]$ of the size $\mathscr{S}_3^*$ of trains (obtained from the activity series of egonetworks centered at each order 3 hyperlink), where a center link is activated at least once, in each collaboration network $\mathscr{H}$ (blue) and its three randomized reference models $\mathscr{H}_3^1$ (yellow), $\mathscr{H}_3^2$ (green) and $\mathscr{H}_3^3$ (red). To identify the trains, we consider $\Delta t = 120d$. For each network, the average size of the trains is reported. The maximum average size among network $\mathscr{H}$, $\mathscr{H}_3^1$, $\mathscr{H}_3^2$ and $\mathscr{H}_3^3$ is in bold. The horizontal and vertical axes are presented in logarithmic scale.

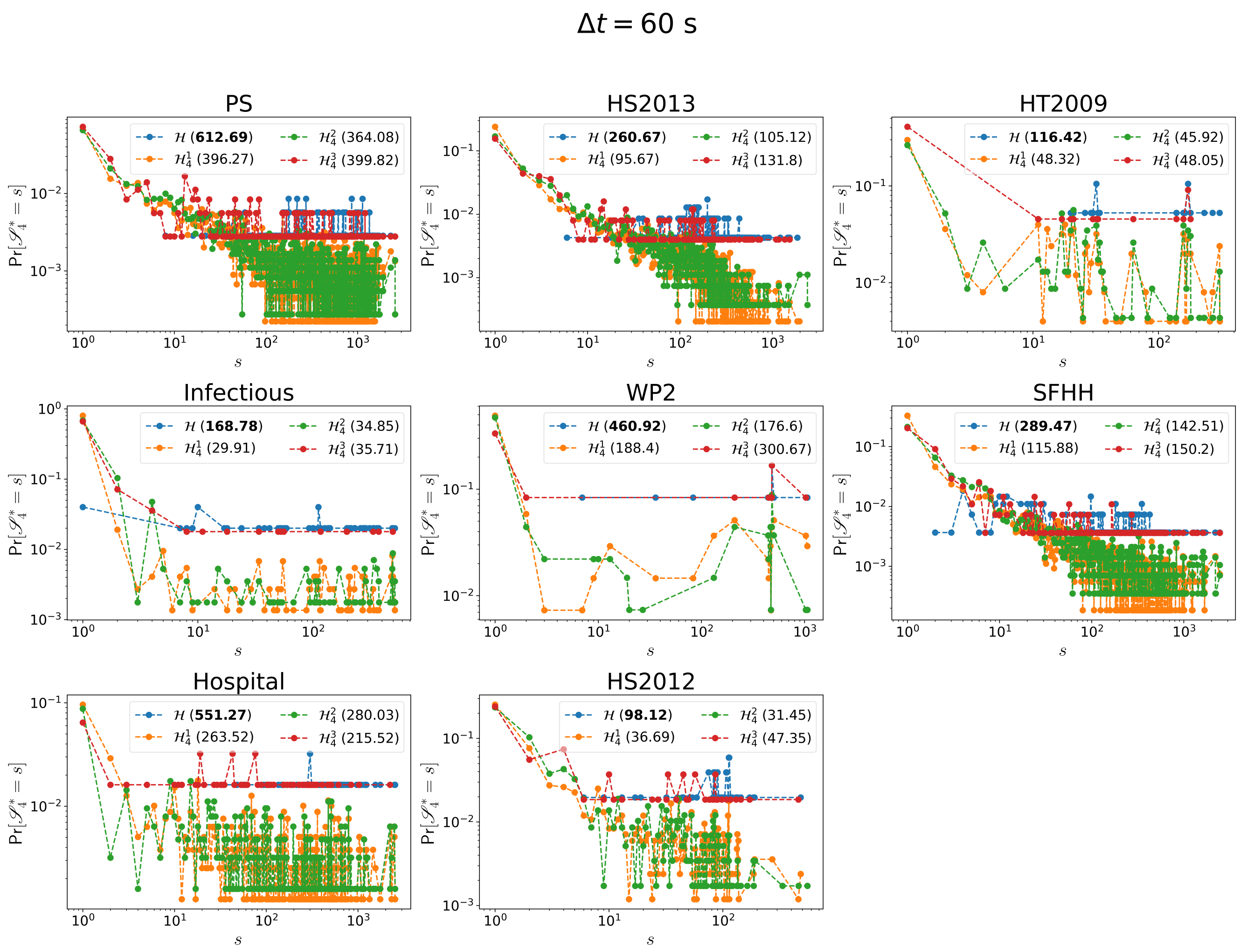

Figure S17. Probability distribution $Pr[\mathscr{S}_4^* = s]$ of the size $\mathscr{S}_4^*$ of trains (obtained from the activity series of egonetworks centered at each order 4 hyperlink), where a center link is activated at least once, in each physical contact network $\mathscr{H}$ (blue) and its three randomized reference models $\mathscr{H}_4^1$ (yellow), $\mathscr{H}_4^2$ (green) and $\mathscr{H}_4^3$ (red). To identify the trains, we consider $\Delta t = 60s$. For each network, the average size of the trains is reported. The maximum average size among network $\mathscr{H}$, $\mathscr{H}_4^1$, $\mathscr{H}_4^2$ and $\mathscr{H}_4^3$ is in bold. The horizontal and vertical axes are presented in logarithmic scale.

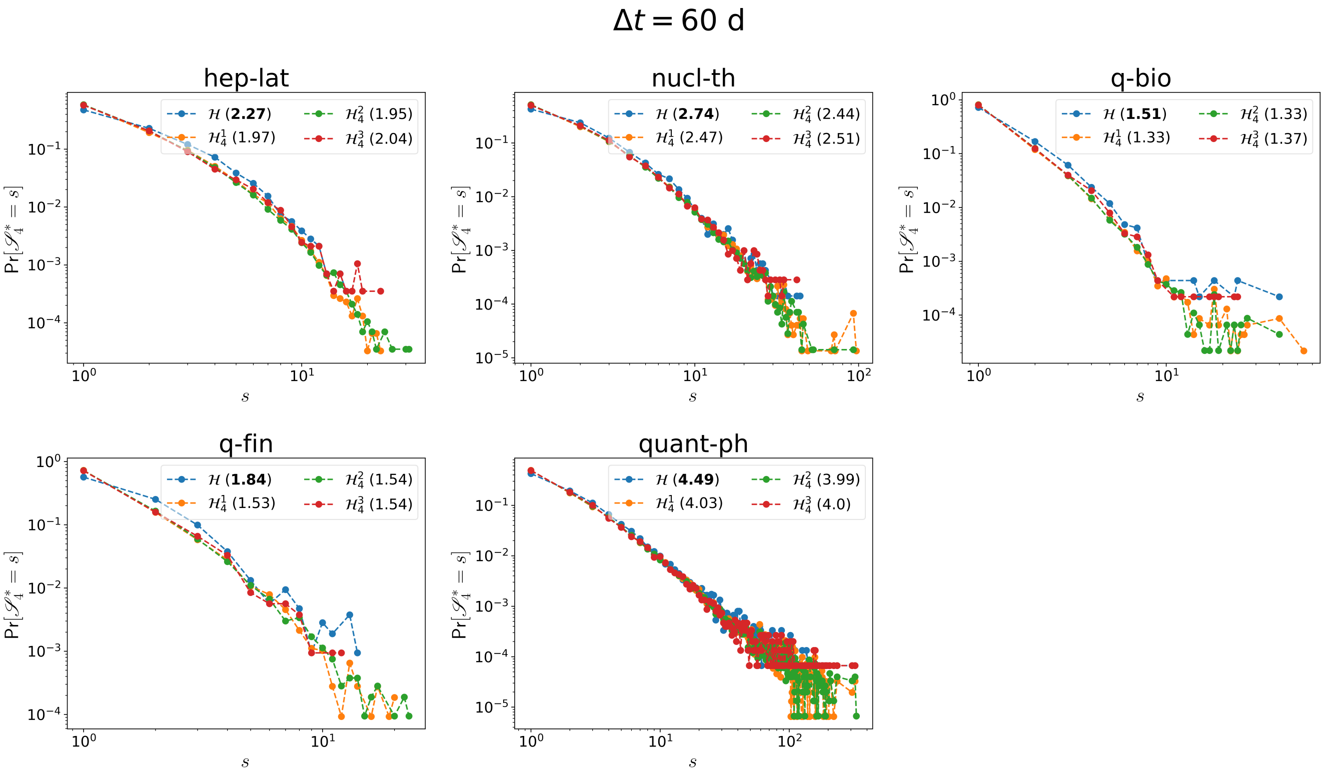

Figure S18. Probability distribution $Pr[\mathscr{S}_4^* = s]$ of the size $\mathscr{S}_4^*$ of trains (obtained from the activity series of egonetworks centered at each order 4 hyperlink), where a center link is activated at least once, in each collaboration network $\mathscr{H}$ (blue) and its three randomized reference models $\mathscr{H}_4^1$ (yellow), $\mathscr{H}_4^2$ (green) and $\mathscr{H}_4^3$ (red). To identify the trains, we consider $\Delta t = 60d$. For each network, the average size of the trains is reported. The maximum average size among network $\mathscr{H}$, $\mathscr{H}_4^1$, $\mathscr{H}_4^2$ and $\mathscr{H}_4^3$ is in bold. The horizontal and vertical axes are presented in logarithmic scale.

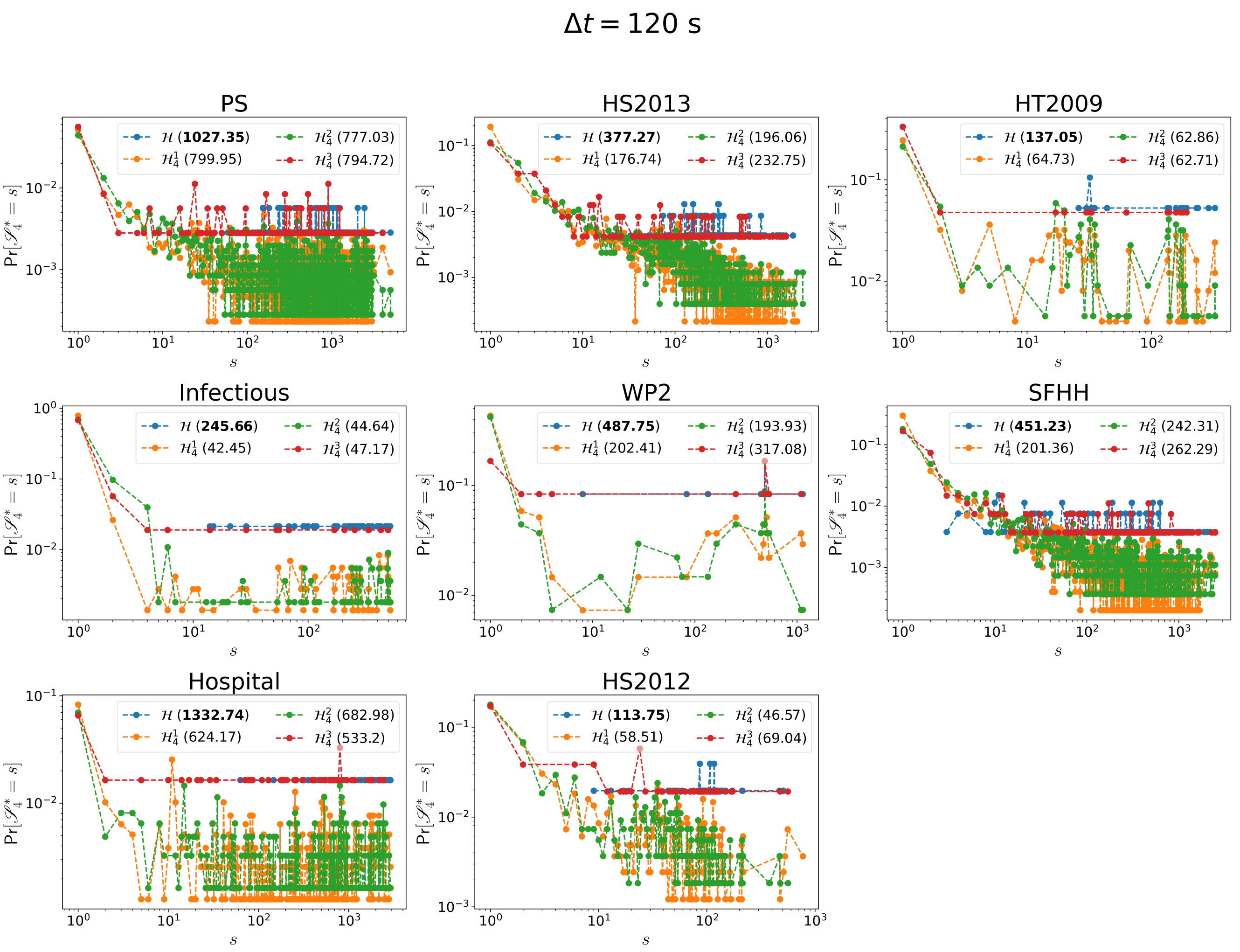

Figure S19. Probability distribution $Pr[\mathscr{S}_4^* = s]$ of the size $\mathscr{S}_4^*$ of trains (obtained from the activity series of egonetworks centered at each order 4 hyperlink), where a center link is activated at least once, in each physical contact network $\mathscr{H}$ (blue) and its three randomized reference models $\mathscr{H}_4^1$ (yellow), $\mathscr{H}_4^2$ (green) and $\mathscr{H}_4^3$ (red). To identify the trains, we consider $\Delta t = 120s$. For each network, the average size of the trains is reported. The maximum average size among network $\mathscr{H}$, $\mathscr{H}_4^1$, $\mathscr{H}_4^2$ and $\mathscr{H}_4^3$ is in bold. The horizontal and vertical axes are presented in logarithmic scale.

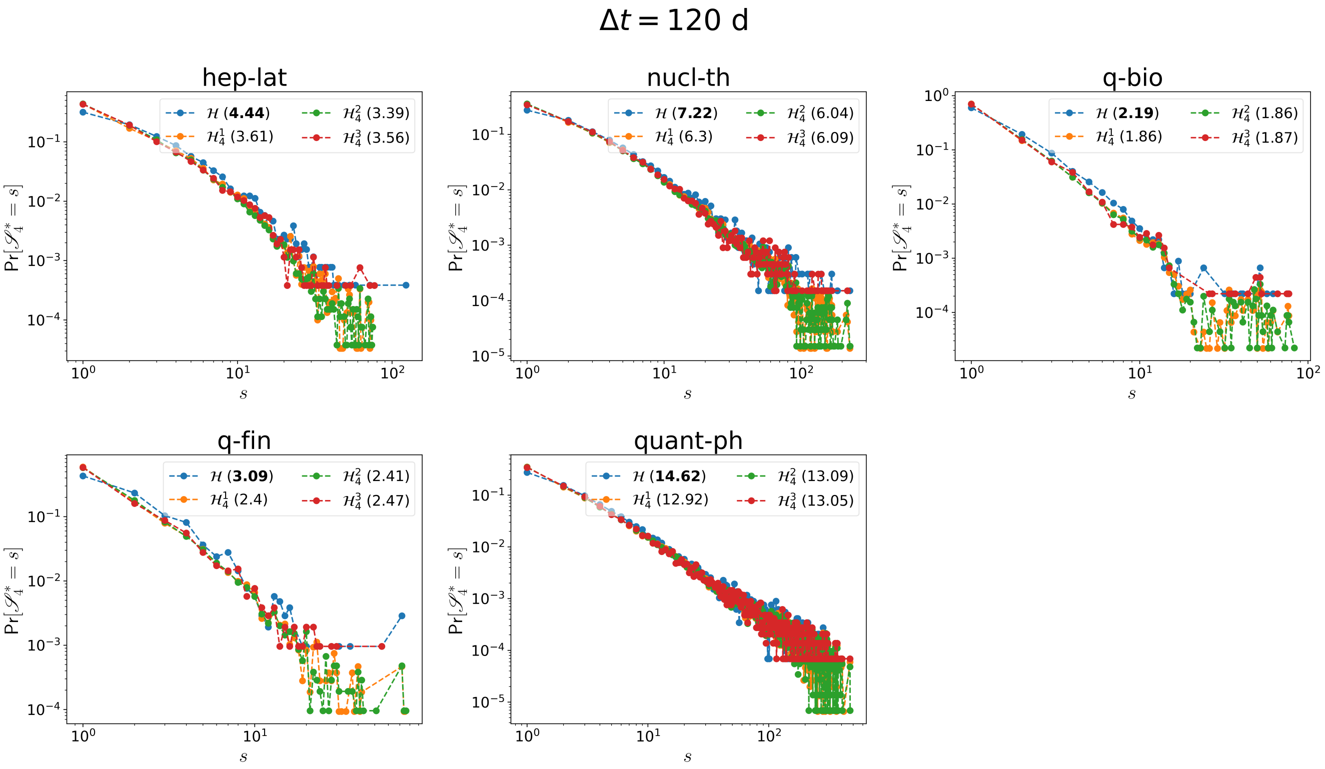

Figure S20. Probability distribution $Pr[\mathscr{S}_4^* = s]$ of the size $\mathscr{S}_4^*$ of trains (obtained from the activity series of egonetworks centered at each order 4 hyperlink), where a center link is activated at least once, in each collaboration network $\mathscr{H}$ (blue) and its three randomized reference models $\mathscr{H}_4^1$ (yellow), $\mathscr{H}_4^2$ (green) and $\mathscr{H}_4^3$ (red). To identify the trains, we consider $\Delta t = 120d$. For each network, the average size of the trains is reported. The maximum average size among network $\mathscr{H}$, $\mathscr{H}_4^1$, $\mathscr{H}_4^2$ and $\mathscr{H}_4^3$ is in bold. The horizontal and vertical axes are presented in logarithmic scale.

C Incomplete higher-order events

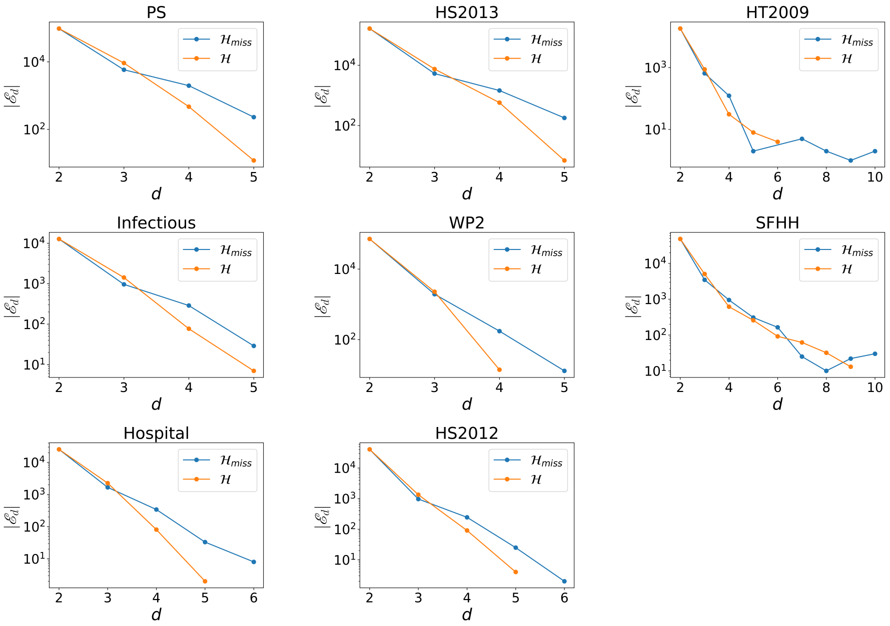

Figure S21. Total number of events ($|\mathscr{E}_d|$) in original network $\mathscr{H}$ and $\mathscr{H}_{miss}$for each order d in physical contact networks. Vertical axis is presented in logarithmic scale.

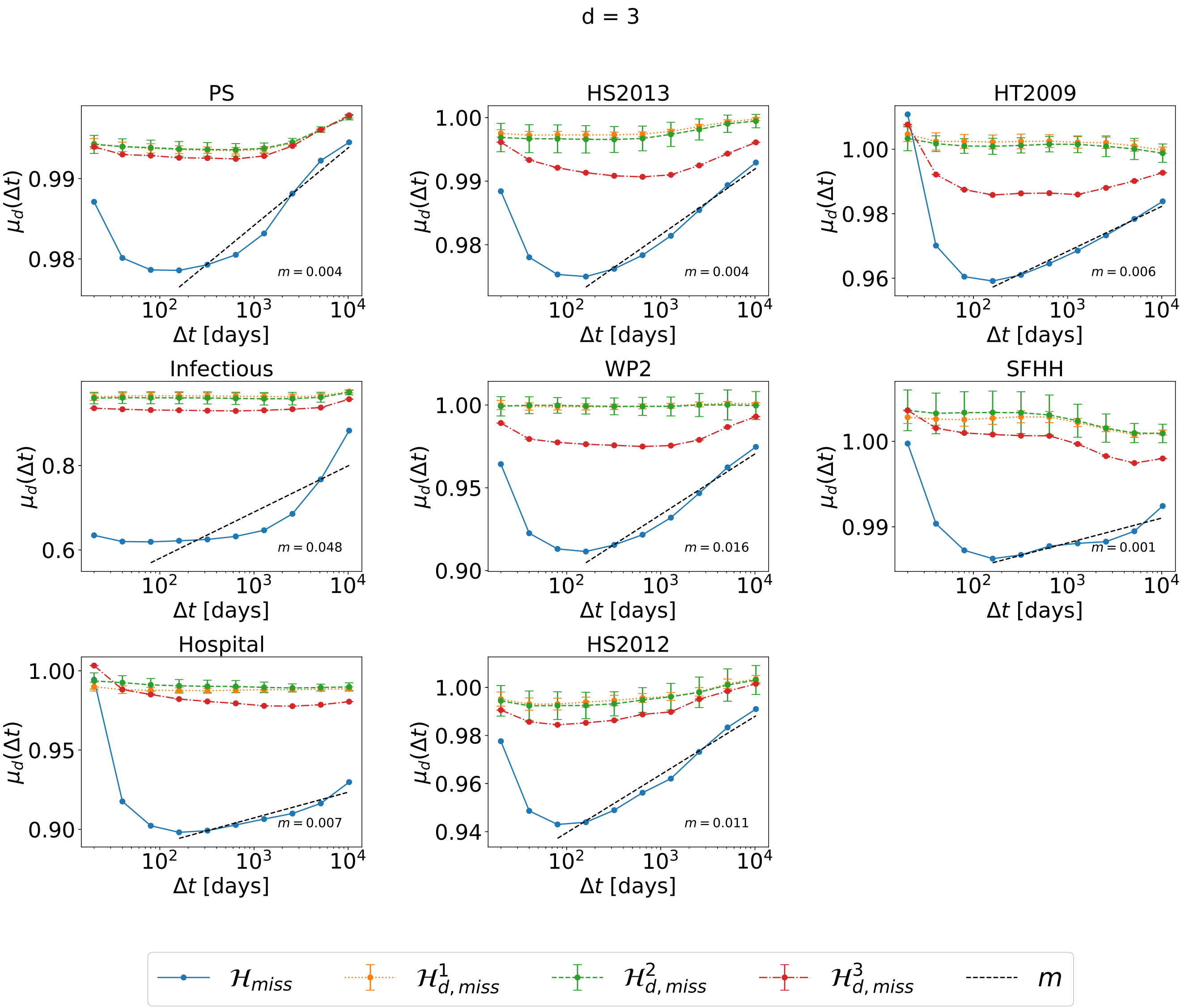

Figure S22. The normalized average topological distance $\mu_d(\Delta t) = \frac{E[\eta(e,e')|\mathscr{T}(e,e')<\Delta t,\ e\in\mathscr{E}_d,\ e'\in\mathscr{E}\setminus\mathscr{E}_d]}{E[\eta(e,e')|\ e\in\mathscr{E}_d,\ e'\in\mathscr{E}\setminus\mathscr{E}_d]}$, between an order $d = 3$ event and an event of a different order, in each physical contact network $\mathscr{H}_{miss}$ and its corresponding three randomized null models $\mathscr{H}^1_{d,miss}$ (yellow), $\mathscr{H}^2_{d,miss}$ (green) and $\mathscr{H}^3_{d,miss}$ (red), which preserve or destroy specific properties of order $d = 3$ events. $\lim_{\Delta t\to\infty} E[\eta(e,e')|\mathscr{T}(e,e') < \Delta t,\ e \in \mathscr{E}_d,\ e' \in \mathscr{E} \setminus \mathscr{E}_d] = E[\eta(e,e')|\ e \in \mathscr{E}_d,\ e' \in \mathscr{E} \setminus \mathscr{E}_d]$ for any d. The horizontal axes are presented in logarithmic scale. The dashed line in each figure corresponds to the linear fit (with slope m) of $\mu_d(\Delta t)$ as a function of $log_{10}(\Delta t)$ in $\mathscr{H}$, for the part that the curve has an increasing trend. For each dataset, the results of the three corresponding randomized models are obtained from 10 independent realizations.

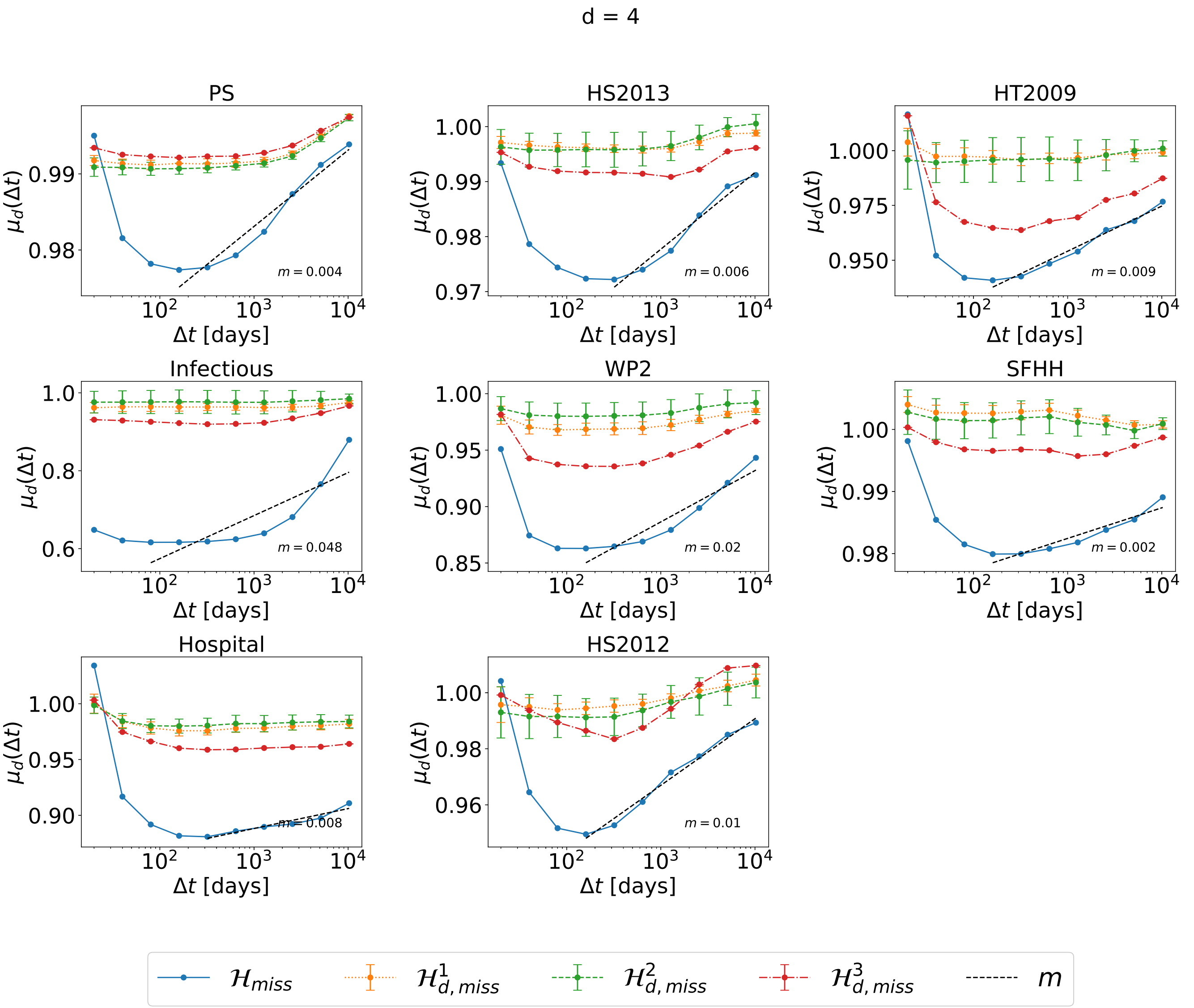

Figure S23. The normalized average topological distance $\mu_d(\Delta t) = \frac{E[\eta(e,e')|\mathscr{T}(e,e')<\Delta t,\ e\in\mathscr{E}_d,\ e'\in\mathscr{E}\setminus\mathscr{E}_d]}{E[\eta(e,e')|\ e\in\mathscr{E}_d,\ e'\in\mathscr{E}\setminus\mathscr{E}_d]}$, between an order $d = 4$ event and an event of a different order, in each physical contact network $\mathscr{H}_{miss}$ and its corresponding three randomized null models $\mathscr{H}^1_{d,miss}$ (yellow), $\mathscr{H}^2_{d,miss}$ (green) and $\mathscr{H}^3_{d,miss}$ (red), which preserve or destroy specific properties of order $d = 3$ events. $\lim_{\Delta t\to\infty} E[\eta(e,e')|\mathscr{T}(e,e') < \Delta t,\ e \in \mathscr{E}_d,\ e' \in \mathscr{E}\setminus\mathscr{E}_d] = E[\eta(e,e')|\ e \in \mathscr{E}_d,\ e' \in \mathscr{E}\setminus\mathscr{E}_d]$ for any d. The horizontal axes are presented in logarithmic scale. The dashed line in each figure correspond to the linear fit (with slope m) of $\mu_d(\Delta t)$ as a function of $log_{10}(\Delta t)$ in $\mathscr{H}$, for the part that the curve has an increasing trend. For each dataset, the results of the three corresponding randomized models are obtained from 10 independent realizations.
